# CRISPR/Cas9 gRNA activity depends on free energy changes and on the target PAM context

**DOI:** 10.1038/s41467-022-30515-0

**Published:** 2022-05-30

**Authors:** Giulia I. Corsi, Kunli Qu, Ferhat Alkan, Xiaoguang Pan, Yonglun Luo, Jan Gorodkin

**Affiliations:** 1 Center for non-coding RNA in Technology and Health, Department of Veterinary and Animal Sciences, University of Copenhagen, Thorvaldsensvej 57, 1871 Frederiksberg, Denmark; 2 Lars Bolund Institute of Regenerative Medicine, Qingdao-Europe Advanced Institute for Life Sciences, BGI-Qingdao, Qingdao, 266555 China; 3 Department of Biology, University of Copenhagen, Copenhagen, 2200 Denmark; 4 Division of Oncogenomics, The Netherlands Cancer Institute, Plesmanlaan 121, 1066 CX Amsterdam, The Netherlands; 5 BGI-Shenzhen, Shenzhen, 518083 China; 6 Department of Biomedicine, Aarhus University, Aarhus, 8000 Denmark; 7 Steno Diabetes Center Aarhus, Aarhus University Hospital, Aarhus, 8200 Denmark

**Keywords:** Computational models, CRISPR-Cas9 genome editing

## Abstract

A major challenge of CRISPR/Cas9-mediated genome engineering is that not all guide RNAs (gRNAs) cleave the DNA efficiently. Although the heterogeneity of gRNA activity is well recognized, the current understanding of how CRISPR/Cas9 activity is regulated remains incomplete. Here, we identify a sweet spot range of binding free energy change for optimal efficiency which largely explains why gRNAs display changes in efficiency at on- and off-target sites, including why gRNAs can cleave an off-target with higher efficiency than the on-target. Using an energy-based model, we show that local gRNA-DNA interactions resulting from Cas9 “sliding” on overlapping protospacer adjacent motifs (PAMs) profoundly impact gRNA activities. Combining the effects of local sliding for a given PAM context with global off-targets allows us to better identify highly specific, and thus efficient, gRNAs. We validate the effects of local sliding on gRNA efficiency using both public data and in-house data generated by measuring SpCas9 cleavage efficiency at 1024 sites designed to cover all possible combinations of 4-nt PAM and context sequences of 4 gRNAs. Our results provide insights into the mechanisms of Cas9-PAM compatibility and cleavage activation, underlining the importance of accounting for local sliding in gRNA design.

## Abstract (web summary)

CRISPR/Cas9-mediated cleavage efficiency varies at different target locations. Here the authors explain this variation with binding free energy changes and show that overlapping Cas9 binding sites influence cleavage efficiency by enabling Cas9 sliding.

## Introduction

The bacterial CRISPR (clustered regularly interspaced short palindromic repeats)-Cas9 endonuclease has been transformed into a powerful genome-editing tool that is nowadays broadly applied in biological, agricultural and medical research^1^. Cleavage by the widely studied *Streptococcus pyogenes* Cas9 ortholog (SpCas9), hereafter referred to as Cas9, is mediated by a 20-nt segment of a guide RNA (gRNA) complementary to a target DNA sequence preceding a protospacer adjacent motif (PAM), where the Cas9 is recruited^2^. The canonical PAM of SpCas9 is 5′- 3′ “NGG”. In this study we refer to this canonical PAM sequence as 5′-N_−1_GGN_+1_-3′, thus including both the GG binding motif and its 1-bp flanking sequence context (N_−1_ and N_+1_) in the definition of PAM. Once a stable gRNA–DNA heteroduplex is formed, double-strand breaks (DSBs) are produced on the DNA by the Cas9 nuclease domains. In the case of the most broadly used SpCas9 protein, DSBs are introduced by the HNH and RuvC nuclease domains 3 nt upstream from the PAM^3^.

A large number of in silico methods for gRNA design report that the cleavage efficiency of Cas9 can largely vary due to the sequence and structural properties of the gRNA^4–17^. However, despite the immense activity in the field, a comprehensive analysis linking gRNA binding patterns, free binding energy changes and unfolding free energy changes to cleavage efficiency is surprisingly still lacking.

A major advance in the understanding of Cas9-gRNA binding was given by Globyte et al., who revealed that DNA interrogation by Cas9 occurs not only by random collisions with the DNA, as previously reported^18^, but also by lateral diffusion. During this process, Cas9 'slides' along the DNA in a local region (≈20 nt)^19^ as part of its search for a target on which the Cas9-gRNA complex can bind firmly. The study of Globyte et al. was, however, limited to the Cas9 sliding dynamics on short-distanced PAMs. Hence, the effect of Cas9 binding at sites overlapping the on-target PAM on cleavage activity remains unexplained.

Previously, we introduced the first energy-based model for Cas9-gRNA-target binding. We applied it to predict Cas9 off-target^20^ and, recently, on-target activities^17^, improving over other available methods. Here, we employ this energy-based model to systematically analyse the relationship between nucleotide bindings and cleavage potential on a large dataset of 11,602 experimentally validated gRNA efficiencies^14,17^. Next, this energy-based model is applied to explain the cleavage activity of Cas9 at gRNA bindings that originate from local overlapping PAMs on which Cas9 slides (local sliding PAMs). Introducing local sliding PAMs in the computation of the CRISPRspec gRNA specificity score, which is a 'competition score' that measures the ability of Cas9 to bind at the on-target while accounting for possible off-targets in the genome, leads to a better identification of gRNAs with high efficiency and low off-target potential. We validated the effect of local sliding PAMs on the cleavage efficiency with public data and in-house generated data from HEK293T cells by altering the sequence and context of Cas9 binding sites at the targets of four gRNAs. Our results further show that local sliding broadens the PAM compatibility of both wild-type and Cas9-derived variants.

## Results

### Cas9 on-target efficiency modelled with binding free energy changes

**Fig. 1:**
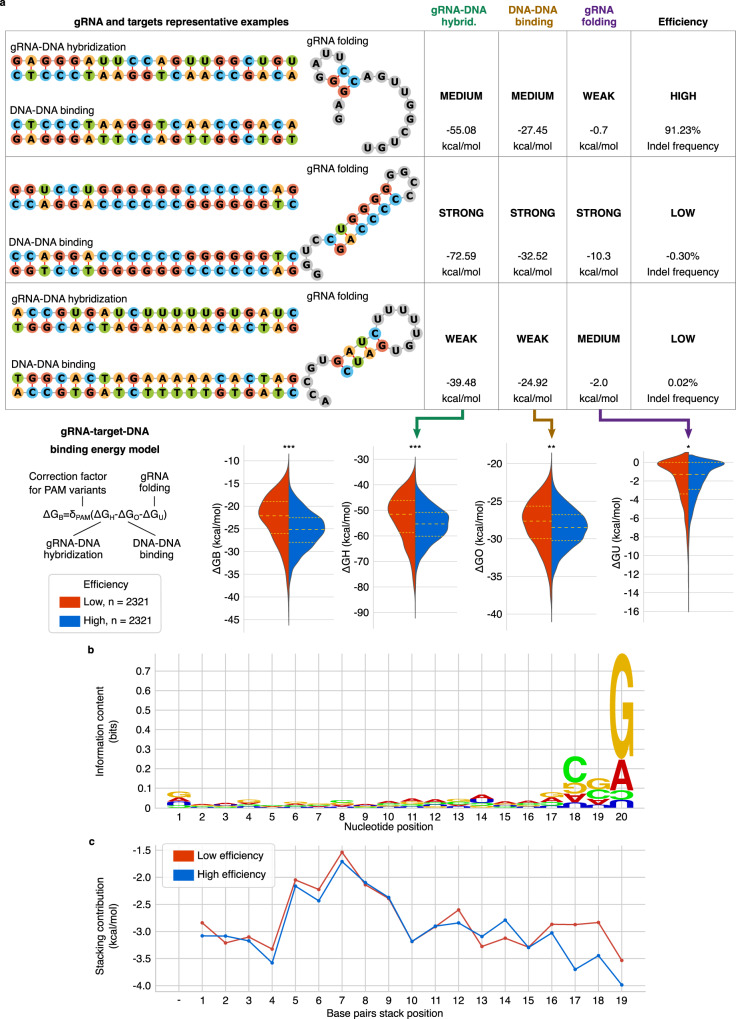
Evaluation of gRNAs free energy change properties and seed preferences. **a** The binding free energy change Δ*G*_*B*_ is dissected in three main components: the RNA–DNA weighted hybridisation free energy change Δ*G*_*H*_, the DNA opening free energy change Δ*G*_*O*_, and the gRNA minimum self-folding free energy change Δ*G*_*U*_. The relationship between these properties and Cas9 efficiency is presented with violin plots comparing highly efficient (top 20%, blue) and inefficient (bottom 20%, red) gRNAs. Statistical significance is computed via the Kolmogorov–Smirnov two-sample test. *P* values (left to right): 7.0E-85 (one-sided), 3.1E-52 (two-sided), 4.0E-20 (two-sided), 4.7E-05 (one-sided). *p < 0.01, **p < 1E-10, ***p < 1E-20. The plots are accompanied by representative examples of gRNA and DNA interactions. **b** Sequence logo of highly efficient gRNAs with position-specific background frequencies extracted from low efficient gRNAs. The sequence logo was generated with slogo^67^ and styled manually. In the logo, bases are shown upside-down when their frequency in the foreground is lower than the expectation derived from the background. **c** Profile of mean base-pair stacking free energy changes in the gRNA–DNA hybrid of gRNAs with high efficiency (top 20%, blue) and low efficiency (bottom 20%, red). The stacking free energy changes are weighted according to previously defined weights estimated from nuclease-dead Cas9 binding kinetics^20,23^. The position of stacking base pairs is parallel to the corresponding nucleotide position in (**b**). Source data are provided as a Source Data file.

Modelling gRNA–DNA bindings in terms of free energy changes is a convenient approach that can be applied to evaluate both on-targets and off-targets with mismatches or bulges. Sufficient gRNA-target-DNA complementarity is essential to trigger the HNH conformational changes that activate DNA cleavage by both HNH and RuvC^21,22^. To test if binding free energy changes can explain this activation we consider the contributions from our previously established CRISPR/Cas9 binding model: $\varDelta {G}_{B}={\delta }_{{PAM}}\left(\varDelta {G}_{H}-\varDelta {G}_{U}-\varDelta {G}_{O}\right)$^20^ with *δ*_*PAM*_ = 1 for 5′-N_−1_GGN_+1_-3′ PAMs. We analysed the relationship between binding free energy changes and on-target cleavage efficiency, measured as indel frequency, in a dataset of 11,602 Cas9 gRNAs binding at targets with 5′-N_−1_GGN_+1_-3′ PAMs from the datasets of Kim et al.^14^ and Xiang et al.^17^, merged following our previously established protocol^17^. During the pre-processing of the original 23,902 gRNAs, instances with low specificity^20^ were removed to avoid biases due to global off-targets competition, and part of the data was isolated as an independent test set for later usage, accounting for data similarity (dataset 'Xiang 2021 test', see Methods and Supplementary Table 1). The gRNA–DNA hybridisation free energy change (Δ*G*_*H*_) is calculated by using stacked gRNA–DNA base pairs weighted based on biochemical profiling of nuclease-dead Cas9 binding kinetics^20,23^. The Δ*G*_*H*_ of highly efficient gRNAs is mostly confined in a sweet spot narrow Δ*G*_*H*_ interval ranging between −64.53 and −47.09 kcal/mol, while more extreme values are observed for inefficient gRNAs (Fig. 1a and Supplementary Table 2). The relation of Δ*G*_*H*_ with the cleavage efficiency is substantially more profound compared to that of the GC-content, despite the high correlation between these two properties (Supplementary Fig. 1). A similar but less pronounced trend emerges for the target DNA–DNA opening free energy change, Δ*G*_*O*_, regarded as a penalty for unwinding the DNA (Fig. 1a and Supplementary Table 3). The gRNA self-folding free energy change, Δ*G*_*U*_, is included in the binding model as a penalty for unfolding gRNA structures, a process required for subsequent target recognition. Our results show that more stable gRNA self-folding structures negatively affect cleavage activity (Fig. 1a and Supplementary Table 4), complementing previous observations^24,25^ within a large-scale scenario. Before binding to Cas9, the scaffold sequence attached to a gRNA can interact with the bases in the gRNA sequence. This can disrupt the optimal structure of the scaffold, whose correct folding is required to form a complex with Cas9^26^. Hence, spacers that interact with the scaffold were removed during data pre-processing whenever the structure of the full gRNA, spacer and scaffold, displayed reduced accessibility of the loops and bulges necessary to bind with Cas9 (see Supplementary Fig. 2 and Methods). The Δ*G*_*B*_ binding free energy change combines Δ*G*_*H*_, Δ*G*_*O*_ and Δ*G*_*U*_, to estimate the residual gRNA–DNA binding free energy change after accounting for the DNA unwinding and gRNA unfolding penalties. The Δ*G*_*B*_ significantly differs between low and high-efficient gRNAs, with the latter obtaining greater benefit in terms of free energy change by binding to the target (Fig. 1a and Supplementary Table 5). However, despite having extremely low (favourable) Δ*G*_*B*_, gRNAs highly rich in GC content remain disadvantageous.

The Δ*G*_*H*_ was further detailed in the position-specific free energy change contributions of stacking gRNA–DNA base pairs and this free energy profile was compared with a sequence logo highlighting the positional nucleotide preferences of highly efficient gRNAs (Fig. 1b, c). We observe that the 3′ seed region of highly efficient gRNAs is characterised by more stable interactions (lower free energy change) with the DNA. When ignoring the estimated impact of Cas9 as the weight on the stacking free energy changes, we notice bigger discrepancies in the 5′ part of the gRNA despite the general lack of sequence variation (Supplementary Fig. 3 and Fig. 1b), supporting that these weights are important in the energy-based model. This profile of free energy changes is consistent with the notion that the differences between efficient and inefficient gRNAs are attributable to the actual binding properties near the PAM, which otherwise are 'only' observable as position-wise single nucleotide variations. The preferred nucleotides in the 3′ seed end of highly efficient gRNAs are guanine at N19-N20 and cytosine at N18-N19, where NX refers to the position in the spacer from the 5′ end. The aversion to uracil (U) at the gRNA 3′ seed end was previously explained as a transcription deficiency, as multiple Ts on the DNA, combined with the downstream T-rich sequence in the DNA sequence of the gRNA scaffold, might trigger transcription termination by polymerase III^27^. Considering that stacking base pairs containing uracil present the lowest binding free energy change benefit (Supplementary Table 6) an additional explanation for this negative effect is possibly the poor hybridisation stability of U-rich gRNA seeds. Supporting this, the presence of up to 2 Us at the gRNA 3′ seed end, not contiguous to the gRNA scaffold and not sufficient to trigger polymerase III termination^28^, is also linked to low efficiency (Supplementary Fig. 4). Altogether, these results reveal that binding and folding interactions between nucleic acids have a significant role in Cas9 cleavage activation, which requires energetically favourable interactions with tight binding at the gRNA 3′ seed region.

### Low gRNA-target hybridisation free energy change results in increased activity at off-targets

In a study investigating the impact of bulges in gRNA–DNA interactions, Lin et al. revealed that DNA bulges are better tolerated at off-target sites with high GC content and that bulged off-targets can even surpass fully complementary interactions in terms of efficiency^29^. To demonstrate this, Lin et al. tested the indel frequency of four gRNAs with different GC content after creating DNA bulges at each position of the target sequences by systematically removing one base at a time in the gRNAs. To explain the efficiency increase recorded at bulged interactions, we examined the affinity between the key free energy change component Δ*G*_*H*_ and the sweet spot. The remaining two components, Δ*G*_*U*_ and Δ*G*_*O*_, presented only minor variations. There were 19 gRNA-target bindings with at least 10% cleavage frequency, which we analysed, i.e., interactions with DNA bulges present at positions at which they are tolerated^29^. The gRNA with the highest GC content (=70%) has an indel frequency of 30% at the fully complementary target, and the Δ*G*_*H*_ of this binding (−66.85 kcal/mol) falls outside of the sweet spot. Strikingly, six bulged off-targets of this gRNA have increased efficiency (indel frequency = 44.77 ± 8.33%) and in five of these the free energy change penalty caused by the bulge (between +6.58 and +15.38 kcal/mol) is sufficient to enter the preferential Δ*G*_*H*_ range (Supplementary Table 7). On the contrary, a gRNA with medium GC content (=50%) and Δ*G*_*H*_ in the sweet spot exhibit lower efficiency in the presence of DNA bulges that push Δ*G*_*H*_ outside of the desired range (Supplementary Table 7). Nothing can be stated for the remaining two gRNAs in the dataset, with 65 and 35% GC content. The former maintains its position in the range of preferential Δ*G*_*H*_ even in the presence of bulges, which reduce the cleavage efficiency. The latter is instead too unstable to tolerate any bulge (high Δ*G*_*H*_).

**Fig. 2:**
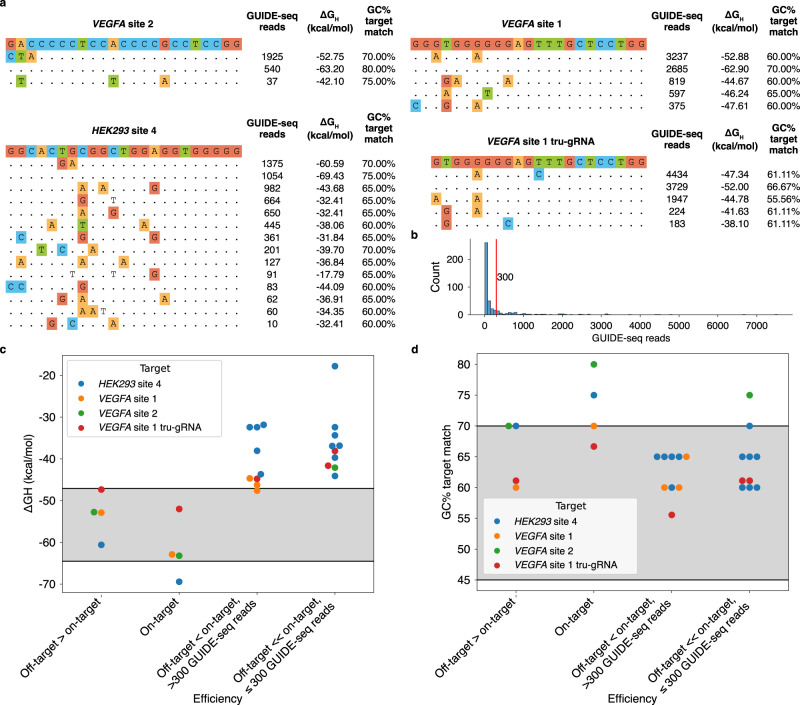
Cleavage activity at off-targets modelled with binding free energy changes. **a** Sequences and properties of off-targets identified by GUIDE-seq^30^ for four gRNAs that have at least one off-target cleaved more efficiently than the on-target. The sequence of the on-target site is reported on top of a list of the profiles of targets detected by GUIDE-seq. Each sequence in the listed targets is shown as follows: dots indicate bases that are the same as the on-target site, while differences between the on-target sequence are displayed with coloured nucleotides. Off-targets were filtered to avoid sites with >3 mismatches to the gRNA, or mismatches located in the four PAM-proximal nucleotides or the PAM itself. **b** Histogram of GUIDE-seq read counts at target sites in the full dataset of Tsai et al. A red vertical bar indicates the arbitrary threshold of 300, used to distinguish sites with low off-target activity. **c** gRNA–DNA hybridisation free energy change Δ*G*_*H*_ of target sites grouped by cleavage activity. The sweet spot preferential range of hybridisation free energy change Δ*G*_*H*_, which contains 80% of the highly efficient gRNAs (top 20% efficient) in the merged dataset of Xiang et al., used for free energy change profiling, is highlighted in grey. **d** Percentage of G and C bases at target positions complementary to the gRNA. Target sites are grouped by cleavage activity. The preferential range of GC%, defined as in (**c**), is highlighted in grey. Source data are provided as a Source Data file.

Inspired by this result, we analysed the GUIDE-seq off-target dataset of Tsai et al.^30^ to understand if mismatches can favour the binding of gRNAs with high GC content by shifting Δ*G*_*H*_ into the sweet spot. Notably, the four gRNAs with the highest GC-content in the dataset (67–80%) have off-targets with mismatches that surpass the on-target in terms of cleavage activity, measured as GUIDE-seq read counts. We focused our analysis on off-targets with no 3-nt PAM (NGG) variation to the on-target and with up to three mismatches to the gRNA, none of which should be in the four PAM-proximal nucleotides, as this could interfere with sequence composition preferences (Fig. 1b). On- and off-target sites (Fig. 2a) were classified by their cleavage activity compared to that of their on-target and by the number of GUIDE-seq read counts, setting a threshold of 300 as low cleavages (Fig. 2b). Of the four on-target sites, three are within the preferential Δ*G*_*H*_ range, while all four off-targets with higher efficiency compared to the on-target reside in the Δ*G*_*H*_ sweet spot. These off-targets are better centered in the preferential Δ*G*_*H*_ range compared to the on-targets, except for the off-target of the gRNA '*VEGFA* site 1 tru-gRNA'. This is a shortened 18-nt gRNA, and as such, it is expected to have higher $\Delta$*G*_*H*_ (less stable binding) compared to the 20-nt gRNAs employed in the rest of the analyses. Instead, all except one of the 18 off-targets with efficiency lower than the on-target fall out of the sweet spot due to their increased Δ*G*_*H*_ (Fig. 2c). Notably, the efficiency at these off-target sites cannot be explained by the GC content of the matching bases at the target site (Fig. 2d). This shows that the position-specific weighted evaluation of matching and mismatching stacking interactions of Δ*G*_*H*_ gives a major benefit in the assessment of gRNA–DNA binding potential compared to a mere nucleotide content calculation.

These results suggest that the sweet spot affinity is a suitable criterion for defining cleavage efficiency at target sites with perfect complementarity, while the combination of the sweet spot range with bulges and mismatches interestingly explains why some off-targets are more efficient than their corresponding on-targets.

### The impact of local sliding over canonical PAMs on cleavage efficiency

**Fig. 3:**
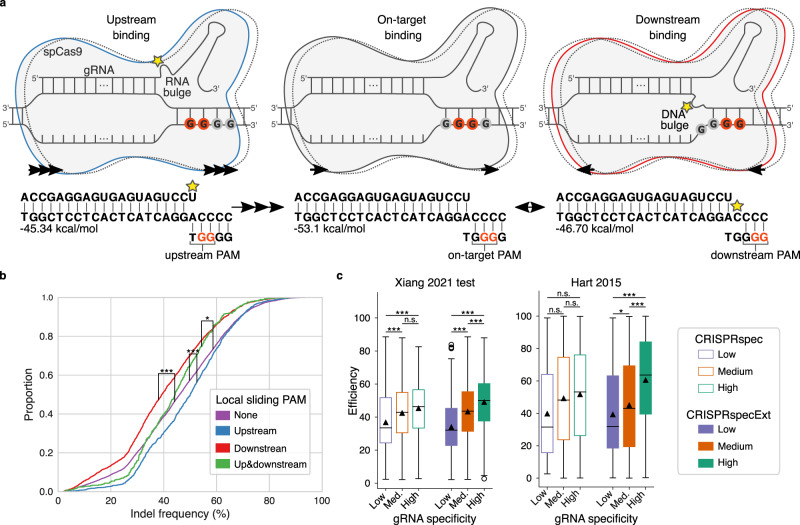
Impact of local sliding PAMs on gRNA efficiency and specificity. **a** Cas9-gRNA binding model in which guanine stretches upstream and downstream from the on-target PAM can function as local sliding PAMs at which the gRNA forms a bulged hybrid with the target DNA. The direction of Cas9 sliding is indicated with arrows, and the upstream and downstream sliding complexes are coloured in blue and red, respectively. Bulges are indicated by yellow stars. Stretches of guanine (G), representing possible PAM binding sites, are reported in the drawing and highlighted in red if used by the complex. **b** Cumulative distributions of indel frequencies of gRNAs categorised by the presence of local sliding PAMs at their targets (one-way ANOVA *p* value = 3.59E-57). Downstream *n* = 2239; upstream *n* = 1339; none *n* = 7565; up and downstream *n* = 459. *T-*test *p* value comparing upstream-none = 1.96E-10 (one-sided), downstream-none = 1.50E-40 (one-sided), up and downstream-none = 3.69E-02 (two-sided). **c** Boxplots of gRNA efficiencies from two datasets binned into low, medium and high CRISPRspec or CRISPRspecExt groups before (left) and after (right) the addition of local sliding contributions. Boxes represent the first and third quartiles (Q1 and Q3). The median is shown as a line and the mean by a triangle; whiskers extend up (or down) to the last (or first) value lower (or greater) than Q3+1.5*(Q3-Q1) (or Q1-1.5*(Q3-Q1)). Number of elements: 'Xiang 2021 test', CRISPRspec low *n* = 204, medium *n* = 1788, high *n* = 227, CRISPRspecExt low = 477, medium = 1377, high = 365; 'Hart 2015', CRISPRspec low = 18, medium = 1019, high = 204, CRISPRspecExt low = 79, medium = 774, high = 388. One-sided Kolmogorov–Smirnov two-sample test, Bonferroni-corrected within each dataset: 'Xiang 2021 test', CRISPRspec low-high = 9.61E-08, medium-high = 0.16, low-medium = 1.04E-07, CRISPRspecExt low-high = 6.63E-27, medium-high = 2.49E-06, low-medium = 3.21E-20; 'Hart 2015', CRISPRspec low-high = 0.06, medium-high = 0.36, low-medium = 0.20, CRISPRspecExt low-high = 1.91E-09, medium-high = 1.07E-12, low-medium = 1.69E-2. *p < 0.05, **p < 0.01, ***p < 0.001. Source data are provided as a Source Data file.

Based on the finding that Cas9 can search for PAMs by sliding on the DNA^19^, we devised an energy-based model to explain the cleavage activity of Cas9 binding at sites with GG motifs overlapping the on-target PAM (Fig. 3a). We reasoned that Cas9 can bind at juxtaposed upstream PAMs forming a gRNA–DNA interaction with a bulge on the first PAM-proximal gRNA nucleotide, and spontaneously slide towards the intended on-target PAM to maximise gRNA–DNA complementarity and increase stability. Conversely, binding at downstream PAMs can anchor the complex in sub-optimal configurations with a DNA bulge between the gRNA–DNA hybrid and the PAM. Except for this single bulge, the hybrid is tightly coupled by 20 base pairs and can thus prevent Cas9 from sliding away or dissociating from the PAM, while keeping the site inaccessible to other Cas9 complexes. However, because a single PAM-proximal DNA bulge can be tolerated^29^, cleavage cannot be excluded at these sub-optimal bindings. Considering the flexibility of nucleic acid bindings, we also do not exclude the possibility of sliding from the on-target PAM to an adjacent downstream PAM at which the gRNA and DNA remain fully bound (Fig. 3a). In line with this model, previous studies have shown that the presence of guanine immediately downstream from the on-target PAM is unfavourable for cleavage efficiency^4,8^. In the merged dataset of Xiang et al. used to study free energy change properties (of 11,602 gRNAs), the mean efficiency at targets with a downstream PAM (DNA bulge) is 12.64% lower compared to the efficiency at targets with isolated non-overlapping PAMs (Fig. 3b). Instead, a mean increase in efficiency of 7.24% characterises targets with upstream PAMs (gRNA bulge). Finally, targets with both down- and upstream sliding PAMs display a percentage decrease in mean efficiency of 3.96%. These observations show that the context of Cas9 binding sites significantly impacts gRNA efficiency.

Multiple overlapping binding sites distanced more than 1 nt from the on-target one while being interspaced from it only by guanines are rare in the dataset (Supplementary Fig. 5). We analysed adjacent PAMs created by G stretches up to 3 nt upstream or downstream of the on-target PAM binding site. The limit of 3 nt is imposed by the size of the target surrogates used to evaluate efficiency, which are integrated randomly in the genome and have unknown context^14,17^. Multiple downstream sliding PAMs do not penalise gRNA efficiency to a greater extent than single ones. Instead, multiple upstream sliding PAMs are more favourable than single ones (Supplementary Fig. 5). However, these involve position −2 or −3 from the on-target binding site (N19, N20), which are part of the seed region, and therefore the increase in efficiency may be related to the nucleotide preferences in the seed in addition to the sliding effects. This bias is not present at sliding PAMs immediately upstream from the on-target binding site (position −1), which do not overlap the gRNA-target. Therefore, in the following, we focus on overlapping PAMs with up to 1 nt from the on-target Cas9 binding site.

Local sliding PAMs allow gRNAs to bind with their targets forming bulged interactions. Bulged bindings are often not accounted for by off-target scoring tools, as their computation is highly demanding. However, the bulged interactions at local sliding PAMs are quick to identify and evaluate, and because of their direct competition with the on-target, they are also highly relevant. Hence, considering bulged interactions at local sliding PAMs as pseudo-off-targets, we extend our off-target scoring to explain how sliding PAMs influence gRNA binding competition. Up- and down-stream local sliding PAMs are integrated into our previously defined CRISPRspec global gRNA specificity score^20^. The higher the CRISPRspec score, the less binding competition (off-targets) affects a gRNA. In general, a score above 5 empirically delineates low off-target potential^20^. In brief, CRISPRspec is calculated as −log_10_ of the fraction between the sum of the Boltzmann-weighted Δ*G*_*B*_ computed at all targets in the genome with up to a certain number of mismatches excluding (numerator) or including (denominator) the on-target^20^. Among competing PAMs, only the most favourable one (lowest Δ*G*_*B*_) is used. Given a gRNA the effect of local sliding PAMs is integrated by adding to CRISPRspec the gain or penalty in efficiency attributable to the local sliding. To do so, CRISPRspec is firstly linearly re-scaled to the efficiency (see Methods). The parameters that define the local sliding addends are obtained by linearly fitting the deviation of the Δ*G*_*B*_ measured at a local sliding PAM from the median Δ*G*_*B*_ computed at all local sliding PAMs of the same type in the dataset (up- or down-stream) to the efficiency. The deviation is used as extreme Δ*G*_*B*_ is unfavourable (Supplementary Fig. 6). To fit the linear model, the merged dataset of Xiang et al. (excluding the test portion) is used with no prior filter on CRISPRspec (14,981 gRNAs, see Supplementary Table 1). The CRISPRspec score extended with local sliding awareness, CRISPRspecExt, shows extensive downgrading and upgrading, respectively, for gRNA targeting sites with downstream and upstream local sliding PAMs (Supplementary Fig. 7). A mixed trend is recorded for gRNA targets that possess both types of local sliding PAMs. Interactions at gRNA targets having neither of the two remain unaffected.

To test if the inclusion of local sliding PAMs improves CRISPRspec, we examined the relation between CRISPRspec and gRNA efficiency before and after the addition of local sliding considerations on two independent test sets. Only gRNAs (20mer) with >3 nt differences to those used for fitting were used (6361 gRNAs for 'Xiang 2021 test', 4026 gRNAs in 'Hart 2015', see Supplementary Table 1). To focus the test on the impact of sliding, the evaluation was performed on gRNAs targeting sites where sliding is possible, i.e., with a downstream or an upstream PAM (2219 gRNAs for 'Xiang 2021 test', 1241 gRNAs in 'Hart 2015'). The gRNAs were partitioned into three equally sized sets of low, medium and high CRISPRspec or CRISPRspecExt. Binning values were estimated on the dataset employed for parameter fitting. In 'Xiang 2021 test' the efficiencies of gRNAs with CRISPRspec medium or high are not different prior to the inclusion of local sliding (Fig. 3c). Other groups, instead, are significantly distinct. After introducing local sliding, the separation between the three groups becomes more profound and all three present significant differences in terms of efficiency (Fig. 3c). On the external independent test set 'Hart 2015', CRISPRspec does not significantly separate gRNAs with different efficiencies, while this task is accomplished by CRISPRspecExt. This analysis shows that CRISPRspecExt can isolate well highly specific, and thus efficient, gRNAs. The positive correlation between our competition score CRISPRspecExt and gRNA efficiency in the 'Xiang 2021 test' and 'Hart 2015' test sets (Pearson’s *r* = 0.29 and *r* = 0.26 respectively) is more substantial compared to that of other relevant properties taken individually, such as the Δ*G*_*B*_ and the GC content (*r* = −0.26 and *r* = 0.15 respectively in 'Xiang 2021 test', no significant correlation in 'Hart 2015', Supplementary Fig. 8). These results support the concept that strong binding competition at local and/or global off-target sites impairs cleavage activity.

### Validation of sliding effects at canonical PAMs in HEK293T cells

**Fig. 4:**
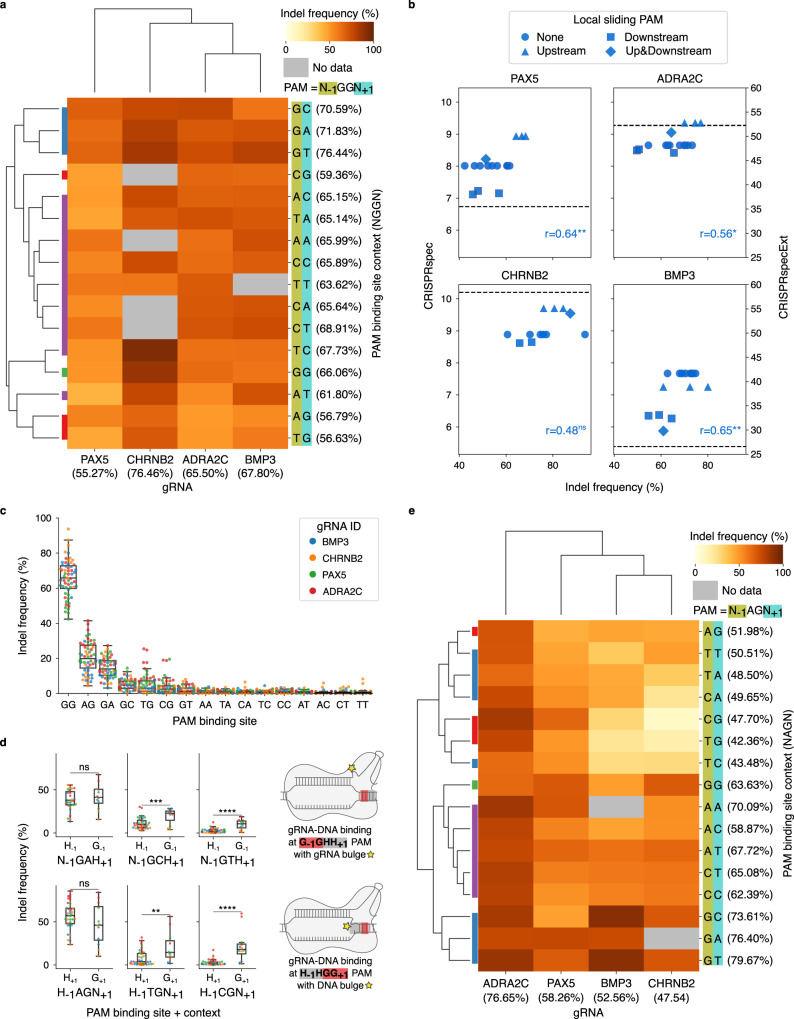
Indel frequency of gRNAs at targets with different PAM sequences in HEK293T cells. **a** Heatmap of indel frequencies (Dox- cells) of four gRNAs targeting sites with 5′-N_−1_GGN_+1_-3′ PAMs clustered by Euclidean distance (missing values were linearly interpolated from targets with the same N_−1_N_+1_ context). Averages of columns and rows are indicated. Leaves in the left dendrogram are coloured as in Fig. 3b. **b** Pearson’s correlation between indel frequency of four gRNAs and their specificity extended with local sliding (CRISPRspecExt, blue colour). *p < 0.05, **p < 0.01, ns non-significant (gRNA PAX5 *p* = 7.05E-03, ADRA2C *p* = 0.02, CHRNB2 *p* = 0.11, BMP3 *p* = 8.73E-3). The specificity score CRISPRspec, not influenced by PAM contexts, is indicated with a dashed line. **c** Indel frequency (Dox- cells) of gRNAs binding at targets with different PAM binding sites (X-axis). The swarm plot details the indel frequencies of each gRNA. Boxes represent the first and third quartiles (Q1 and Q3). The median is shown as a line; whiskers extend up (or down) to the last (or first) value lower (or greater) than Q3+1.5*(Q3-Q1) (or Q1-1.5*(Q3-Q1)). Number of gRNAs per box, left to right: 59; 56; 53; 56; 60; 54; 62; 60; 58; 61; 59; 62; 58; 57; 62; 63. **d** Indel frequency (Dox+ cells) at targets with alternative PAMs but canonical GG local sliding PAM upstream (top) or downstream (bottom). Boxes and swarm plots are defined as in (**c**). Binding contexts represented in less than three of four gRNAs or with G_+1_ (top) or G_−1_ (bottom) were excluded. See Supplementary Figs. 12–15 for results on all PAMs and cell treatments. One-sided *t*-test *p* values from left to right, top to bottom: 0.17; 9.72E-04; 1.69E-07; 0.94; 1.40E-03; 2.59E-07 (*p < 0.05, **p < 0.01, ***p < 0.001, ****p < 1E-04). The alternative hypothesis is that efficiencies at targets with G_−1_ (top) or G_+1_ (bottom) have a larger mean than, respectively, H_−1_ or H_+1_. Number of elements per box, from left to right, top to bottom: 30; 12; 35; 11; 36; 11; 35; 12; 36; 11; 35; 12. **e** Heatmap of indel frequencies (Dox+ cells) of four gRNAs at targets with 5′-N_-1_AGN_+1_-3′ PAMs. See (**a**) for further details. Source data are provided as a Source Data file.

As revealed from the analysis above, the gRNA efficiency is affected by the context flanking the on-target PAM binding site. To further validate this effect, we measured the efficiency of four gRNAs for the genes *PAX5*, *BMP3*, *ADRA2C* and *CHRNB2*. These gRNAs displayed high cleavage efficiency in our previous surrogate-based evaluation of gRNAs and exhibited no trace of enrichment or depletion in the edited cells^17^ (see Methods). For each gRNA, target DNA surrogate sites were designed to carry all possible 16 variations of N_−1_ and N_+1_ in the 5′-N_−1_GGN_+1_-3′ PAM (Supplementary Table 8). The efficiency was measured as indel frequency 6 and 10 days after lentiviral transduction in HEK293T cells, following our previously established protocol^17^ (see Methods). The efficiencies obtained at the two time points were strongly correlated (Pearson’s *r* = 0.98, Spearman’s *R* = 0.79) and were therefore averaged (Supplementary Fig. 9 and Supplementary Data 1). In agreement with the local PAM-sliding model presented above, the cleavage at targets with G_−1_GGH_+1_ and H_−1_GGG_+1_ PAMs displays, in this order, percentage increase and decrease in mean efficiency of 11.31 and 12.13% compared to sites with H_−1_GGH_+1_ PAM, where H symbolises any of A, T or C. Clustering targets based on the N_−1_N_+1_ context produces an exclusive cluster for G_−1_H_+1_, which comprises the top three contexts with highest mean efficiency across the four gRNAs, while the lowest 3 belong to the H_−1_G_+1_ contexts (Fig. 4a).

If local sliding PAMs are not considered, CRISPRspec is constant for different PAM contexts of the gRNA targets. In contrast, CRISPRspecExt is positively correlated to the gRNA efficiency, thanks to the addition of the sliding PAM contributions (Fig. 4b). Thus, our energy-based binding model allows improved identification of highly specific and efficient gRNAs based on the context of their targets. The portion of the variance associated with the differences in the PAM binding site context explained by the sliding activity on adjacent canonical 'GG' binding motifs was assessed as the sum of squares to the grand mean relative to each condition (sliding upstream, downstream, on both sides or on none) and weighted by the size of each group, divided by the total sum of squares (see Methods). The sliding activity explains >50% of the variance in the targets designed for the gRNAs ADRA2C (53.49%), PAX5 (52.44%) and BMP3 (50.58%) while this proportion is lower, 32.44%, in the case of gRNA CHRNB2. Other features of the target sites may be responsible for the remaining, unexplained, variance. For instance, the random integration of the surrogate sequences carrying the targets may impact their accessibility and, therefore, the ability of Cas9 to cleave these sites, which is a limitation of the current surrogate-based approaches^17^. In addition, the effect of the different context nucleotides on PAM-probing and the possible sliding activity of Cas9 on non-canonical PAMs, described in the next section, may also contribute to the remaining variance (i.e., binding and sliding at G_−1_GGH_+1_ may variate for different H_+1_). Additional data will be necessary to evaluate this feature and eventually include it in our sliding model.

### Local sliding broadens non-canonical PAM compatibility

Cleavage by Cas9 can also verify at target sites flanked by non-canonical PAMs, of which N_−1_AGN_+1_ is reported as the most active^10^. To test if the concept of local sliding is applicable to non-canonical PAMs, we evaluated the efficiency, measured as indel frequency, of the same four gRNAs described above for target DNA sites bearing all possible variations of the PAM binding motif and its context. The activity of Cas9 at non-canonical PAMs is generally low, therefore we additionally measured gRNA efficiency in HEK293T cells with Doxycycline-induced overexpression of Cas9 (Dox+). The gRNA efficiencies measured in Dox+ and Dox− cells are well correlated (Supplementary Fig. 10). In untreated cells (Doxo−) the mean efficiency at targets flanked by the canonical GG PAM binding site is 65.49% while at the non-canonical AG and GA it is respectively 21.18 and 14.93%. At other non-canonical binding sites, the mean efficiency is below 10% (TG = 5.29%, GC = 5.06%, CG = 3.69%, GT = 2.00%, AA = 1.12%, TA = 0.90%, CC = 0.88%, CA = 0.85%, TC = 0.81%, TT = 0.72%, AT = 0.71%, CT = 0.62%, AC = 0.61%) (Fig. 4c). In the Dox+ group (Supplementary Fig. 11), extremely high efficiency is obtained at bindings with a canonical PAM regardless of their context (mean = 96.79%). The efficiency at the non-canonical AG binding site in Dox+ reaches levels close to those of GG in the Dox− group (mean = 59.54%). The overexpression of Cas9 increases the efficiency at sites flanked by other non-canonical PAMs as well, such as those with binding sites GA, TG, GC and CG, with mean efficiencies of 41.50, 15.09, 12.75 and 8.86%, respectively. Cleavage at targets with other PAMs remains instead rare or null (mean efficiency <5% for all).

Among all possible alternative PAMs those with at least one G in the binding motif are the most efficient ones (Fig. 4c). These sites can incorporate local sliding PAMs with a canonical GG binding motif if a G is present in the context upstream (G_−1_GNN_+1_) or downstream (N_−1_NGG_+1_). Following Cas9 binding at such PAMs, gRNAs may form bulged interactions with their targets in lack or excess of 1 nt (Fig. 4d). Indeed, the cleavage efficiency measured in Dox+ HEK293T cells at alternative PAMs with a canonical GG binding motif upstream (G_−1_GCH_+1_ and G_−1_GTH_+1_) or downstream (H_−1_TGG_+1_ and H_−1_CGG_+1_) is significantly higher compared to that of targets with the same alternative PAM binding sites but no G in the N_−1_N_+1_ context (Fig. 4d, see also Supplementary Figs. 12–15 for results on all PAM binding sites in Dox- and Dox+). Alternative PAMs with binding motif AG and GA, which are the closest to the canonical GG in terms of efficiency, do not display a preference for local sliding toward canonical PAMs. This suggests that fully complementary gRNA–DNA interactions at a non-canonical AG or GA are equally tolerated as bulged bindings at the canonical GG PAM binding site.

The efficiencies related to target sites with G_−1_AGH_+1_ PAMs in Dox+ cells are significantly more efficient (mean increase 33.45%) compared to H_−1_AGH_+1_ PAMs (Fig. 4e, Supplementary Fig. 14). This trend is similar to the one observed for the canonical G_−1_GGH_+1_ PAM in Dox−. G_−1_ generates a non-canonical GA binding site, less stable than GG or AG. This could facilitate further the search of Cas9 for a more stable binding site, both in terms of protein and gRNA binding. Conversely, the change in mean efficiency (decrease of 17.47%) registered at H_−1_AGG_+1_ compared to H_−1_AGH_+1_ is non-significant (Supplementary Fig. 15). G_+1_ locks Cas9 at a GG binding site, more favourable than AG, but with 1 nt DNA bulge between the PAM and the gRNA–DNA hybrid. Although less pronounced, these effects are also visible in the Dox− group (Supplementary Fig. 16). Similarly, no significant change in efficiency is observed at G_−1_GAH_+1_ (increase in mean efficiency = 10.10%) compared to H_−1_GAH_+1_ (Supplementary Figs. 14, 16). In this case, G_−1_ produces a canonical upstream GG binding site that may antagonise the attempt of Cas9 to maximise the gRNA–DNA binding stability, as this requires sliding towards a less favourable PAM (GA binding site). The possible gRNA–DNA hybrid formed at this site contains a 1-nt gRNA bulge and therefore has limited efficiency. Instead, H_−1_GAG_+1_ PAMs have an increased mean efficiency by 22.22% over H_−1_GAH_+_ (Supplementary Fig. 15). Downstream G_+1_ creates a local PAM sliding with AG binding site, which does not anchor Cas9 as in the previous cases (GG binding site) but may favour the search for a different PAM in the neighbourhood.

### Evidence for local sliding in Cas9 variants

**Fig. 5:**
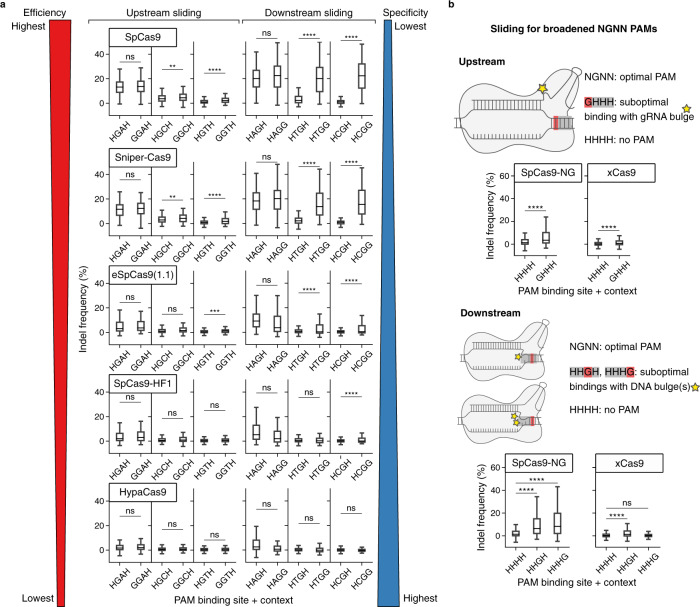
Sliding affects PAM recognition by Cas9 variants. **a** Boxplots of indel frequencies produced by Cas9 variants in HEK293T cells at targets followed by different PAMs (X-axis). Each row represents a Cas9 variant, sorted from top to bottom by efficiency and inverse specificity. Boxplots are separated in two groups for upstream (left) and downstream (right) sliding. Boxes represent the first and third quartiles (Q1 and Q3). The median is shown as a line; whiskers extend up (or down) to the last (or first) value lower (or greater) than Q3+1.5*(Q3-Q1) (or Q1-1.5*(Q3-Q1)). One-sided *t*-test *p* values, Bonferroni-corrected by the number of Cas9 variants: from left to right, top to bottom: 1.00; 4.90E-03; 4.15E-09; 0.06; 9.60E-49; 5.53E-84; 0.83; 8.44E-03; 2.43E-07; 0.40; 1.03E-39; 1.78E-63; 1.00; 0.09; 6.87E-04; 1.00; 5.54E-06; 1.08E-12; 0.85; 0.32; 0.13; 1.00; 0.078; 1.16E-05; 0.71; 0.71; 0.32; 1.00; 0.59; 0.06. The alternative hypothesis is that gRNA efficiencies at targets with G_−1_ (left) or G_+1_ (right) have larger mean, respectively, than targets with H_−1_ or H_+1_. Number of elements in each box, from left to right, top to bottom: 265; 88; 261; 85; 267; 85; 263; 88; 260; 86; 265; 86; 265; 88; 262; 86; 267; 85; 264; 88; 260; 87; 265; 86; 265; 88; 260; 86; 267; 85; 264; 88; 259; 87; 265; 86; 265; 88; 262; 86; 267; 85; 265; 88; 260; 87; 265; 86; 265; 88; 262; 86; 266; 85; 264; 88; 260; 87; 264; 86. **b** Illustration of the sliding mechanism at N_−1_GNN_+1_ PAMs, and boxplots of indel frequencies in HEK293T cells produced by Cas9 variants with broadened PAM compatibility at targets with different PAMs (X-axis). Boxes are defined as in (**a**). One-sided *t*-test *p* values, Bonferroni-corrected by the number of Cas9 variants: from left to right, top to bottom: 2.30E-59; 8.68E-25; 2.12E-135; 1.94E-186; 7.06E-69; 1.00. The alternative hypothesis used is that gRNA efficiencies at GHHH (upstream case) or HHGH/HHHG (downstream case) targets have larger mean than at HHHH. Number of elements in each box, from left to right, top to bottom: 2322; 799; 2317; 778; 2322; 786; 772; 2317; 786; 770. Source data are provided as a Source Data file.

To corroborate our observations on the local sliding activity of Cas9, we analysed the data from Kim et al.^16^, which measured indel frequencies of gRNAs using 13 SpCas9 variants^16^, consisting of the wild-type SpCas9, five high-fidelity variants (eSpCas9(1.1)^31^, SpCas9-HF1^32^, HypaCas9^33^, evoCas9^34^ and Sniper-Cas9^35^), five variants with altered PAM compatibility (VQR^36^, VRER^36^, VRQR^32^, QQR1^37^ and SpCas9-NG^38^), and two variants with both such properties (VRQR-HF1^32^ and xCas9^39^). We and others previously reported that the data presented in Kim et al.^16^, in which a modified gRNA scaffold is employed, is not entirely compatible with that of similar recent studies^17,40^. A closer look into the library dedicated to the analysis of PAM contexts (for which cleavage at surrogate targets followed by all combinations of 4-nt PAMs was examined for 30 gRNAs) reveals that in the case of SpCas9, gRNAs with a U at the 3′ seed end (U20) are more efficient (indel frequency mean ± std = 52.46 ± 5.44%, *n* = 110 targets of seven gRNAs) compared to gRNAs with a C20 (mean ± std = 46.23 ± 6.90%, *n* = 239 targets of 15 gRNAs) or an A20 (mean ± std = 47.44 ± 7.64%, *n* = 121 targets of eight gRNAs) (two-sided *t-test* *p* value C20 compared to U20 = 1.66E-15, A20 compared to U20 = 3.61E-08). Of note, no gRNA with a G20 is present in the dataset. The higher efficiency observed in gRNAs with a U20 is in contrast with our results of nucleotide preferences based on the merged data from Kim et al.^14^ and Xiang et al.^17^ (Fig. 1b), as well as with other independent reports^4,8,15^. The nucleotide N20 constitutes the gRNA bulge in the upstream sliding and thus plays a central role in the analysis of sliding dynamics. Because of this incongruence, we do not validate local sliding on the canonical PAM using the Kim et al.^16^ dataset. The PAM preferences in such dataset are, instead, as expected, with GG, AG and GA being the preferred binding sites (in this order), and other PAM binding sites showing little or no activity (Supplementary Fig. 17). As the efficiency variation linked to different PAM binding sites is much higher compared to that of sliding dynamics on canonical PAMs, we can reliably use the dataset from Kim et al.^16^ to further explore our hypothesis on Cas9 sliding from non-canonical toward adjacent canonical PAMs. In the case of the wild-type SpCas9, targets followed by alternative PAMs G_−1_GCH_+1_, G_−1_GTH_+1_ are cleaved more efficiently compared to targets with the same alternative binding site but no G at position −1 (Fig. 5a). Similarly, targets followed by alternative PAMs H_−1_TGG_+1_ and H_−1_CGG_+1_ are cleaved more efficiently than H_−1_TGH_+1_ and H_−1_CGH_+1_ (Fig. 5a). No increase in efficiency is observed for the PAMs G_−1_GAH_+1_ and H_−1_AGG_+1_ compared to H_−1_GAH_+1_ and H_−1_AGG_+1_, due to the higher efficiency at these alternative PAM binding sites (Fig. 5a). These results are congruent with those obtained with the data we generated. Moreover, we examined high-fidelity Cas9 variants that do not contain variations in the PAM-recognition domains after sorting them by efficiency and inverse specificity, previously assessed^16^. The same preferences for up- and down-stream sliding toward canonical PAMs are visible in Sniper-Cas9, which is the variant closest to the wild-type in terms of efficiency and specificity, and gradually disappear in more specific variants (eSPCas9(1.1), SpCas9-HF1, HypaCas9), less tolerant to imperfect gRNA–DNA bindings (Fig. 5a). The variant evoCas9 was shown to be generally poorly active^16^ and was thus excluded.

Furthermore, we analysed the efficiency of the Cas9 variant SpCas9-NG, which shows good compatibility with N_−1_GNN_+1_ PAMs, and xCas9, which has increased binding specificity and tolerates N_−1_GNN_+1_ PAMs, although N_−1_GGN_+_1 remains highly preferred^16^. In these variants, upstream sliding is expected at G_−1_HHH_+1_ PAMs, while H_−1_HGH_+1_ and H_−1_HHG_+1_ may result in downstream sliding of, respectively, 1 or 2 nucleotides (Fig. 5b). Consistently with our model, targets followed by GHHH are cleaved more efficiently than those followed by HHHH, in both variants (Fig. 5b top). Downstream sliding shows increased efficiency at targets with either of the PAMs HHGH and HHHG compared to HHHH in SpCas9-NG, while in xCas9 only HHGH is more efficient than HHHH (Fig. 5b bottom). This is expected considering the increased specificity of xCas9, that may tolerate one but not two DNA bulges between the gRNA–DNA binding and the xCas9 PAM binding site. The consistent efficiency increase observed at PAMs that allow for up- or downstream sliding to canonical binding sites and the gradual decrease of this effect in the most specific Cas9 variants strongly support our model for broadened PAM compatibility enabled by sliding on adjacent PAMs.

## Discussion

In this work, we describe CRISPR/Cas9 cleavage as an energy-driven process in which efficiency significantly depends on nucleotide hybridisation and folding free energy changes. Our analysis of the free energy change preferences of efficient and inefficient gRNAs underlines the importance of designing gRNAs with low self-folding stability, balanced hybridisation of free energy change and firm target binding at the seed region. In regard to the ongoing discussion on whether Cas9 binding can be modelled in equilibrium^41^, here we show that this is the case. Notably, our sweet spot of gRNA–DNA hybridisation free energy change provides an explanation for the higher cleavage activity previously measured at bulged or imperfectly matching off-targets compared to on-targets. Recent studies report that the mismatch tolerance patterns of different gRNAs can highly variate^42^, and that sequence composition features such as enrichment of guanine and depletion of uracil characterise gRNAs with high guide-intrinsic mismatch permissiveness^43^. Our results suggest that the fitness to the sweet spot and the free energy changes related to imperfect matches to the gRNA are suitable criteria to evaluate guide-intrinsic mismatch tolerance. By expanding the concept of gRNA-target-DNA hybridisation with the inclusion of imperfect matches containing gRNA or DNA bulges, we explain the activity of gRNAs at targets with adjacent PAMs on which Cas9 can locally slide. In the merged dataset of Xiang et al. used to examine binding free energy change properties, such local sliding activity affects ≈35% of the gRNAs that have a target site in hg38. We show that sliding activities can affect cleavage efficiency both positively and negatively, depending on the binding free energy change at bulged interactions. The inclusion of local sliding effects, which can be interpreted as local off-targets, improves the definition of gRNA specificity, previously based solely on global off-targets. Although interactions at sliding PAMs can result in DNA cleavage and produce indels near the target site, fulfilling the editing purpose in knockout experiments, their activity nearby the on-target site can lead to undesired effects in applications that require Cas9 to be positioned precisely at the target, such as in base editing. We validate the effect of local sliding on gRNA cleavage efficiency by analysing independent data (Fig. 3b, c) and by generating efficiency data for 4 gRNAs targeting DNA sites that carry all possible alterations of the Cas9 binding site and its immediate context (Fig. 4 and Supplementary Figs. 12–16). Such evaluation of different PAMs and contexts for the same gRNAs eliminates the bias that would derive from measuring the effect of contexts and PAMs using distinct gRNAs. Furthermore, we reveal that cleavage can be obtained more efficiently at targets followed by non-canonical PAMs whenever sliding on adjacent canonical PAMs is possible. This result, supported both by our data and by public gRNA efficiency data from wild-type and engineered Cas9 variants, implies that local sliding broadens Cas9-PAM compatibility. This can be highly useful in those situations in which the choice of sites to be targeted for cleavage is limited (e.g., knock-in of genomic variants). While our study is centred on Cas9-gRNA interactions, the concepts we describe can be extensively applied in relation to other RNA-guided complexes. In conclusion, the local context of Cas9 binding sites can strongly impact Cas9 binding and cleavage efficiency, and this can largely be explained by our binding energy-based model. We also show that the assessment of gRNA specificity is enhanced once local sliding PAMs are considered and that this helps to identify gRNAs with higher specificity and efficiency.

## Methods

### Cell culture

Human embryonic kidney cells (HEK293T, originally purchased from ATCC catalogue num. CRL-3216) were cultured in DMEM media containing 10% foetal bovine serum (FBS) and 1% penicillin–streptomycin in a tissue culture incubator at 37 °C with 5% CO_2_. PCR mycoplasma detection kit (catalogue num. PM008, Shanghai Yise Medical Technology) was routinely used to test the mycoplasma contamination. The cells used in this study gave negative results in the mycoplasma contamination test. SpCas9-expressing HEK293T (HEK293T-SpCas9) cells were generated by a PiggyBac transposon system followed by selection in the presence of 50 µg/ml hygromycin to ensure high Cas9 activity. HEK293T cells were transiently transduced with pPB-TRE-spCas9-Hygromycin vector and pCMV-hybase vector with a 9:1 ratio to generate SpCas9-expressing HEK293T.

### Surrogate library design and plasmid library preparation

To construct the gRNA-target-DNA surrogate library we selected four gRNAs with indel efficiency >90% in our previous CRISPRon chip (Dox+)^17^ and designed complementary targets flanked by 4-nt PAM sites variated in all possible ways (PAM=NNNN). This resulted in 4^4^ = 256 gRNA-target-DNA surrogates, plus 10 positive sequence surrogates. The total number of sequences we generated was 1062 because two sequences containing BsmBI restriction sites were filtered out. Oligos were structured as follows: 20 bp gRNA, 82 bp gRNA scaffold, 37 bp surrogate target (10 bp barcode, 20 bp protospacer, 4 bp random PAM, 3 bp downstream sequence).

The 1062 PAM library oligo was synthesised in Genscript^®^ (Nanjing, China). All sgRNA sequences and their oligos are listed in Supplementary Data 2. The library was cloned as a pool into a LentiU6-LacZ-GFP-Puro (BB) lentiviral plasmid (Addgene ID:170459) for lentivirus production as previously described in ref. ^17^. Briefly, plasmid library cloning started with PCR amplification of the 170-nt oligonucleotide pool. Firstly, the library oligos were diluted to 1 ng/µl and then PCR amplification was performed using the primers: TRAP-oligo (BsmBI GGA)-F(5′-TACAGCTaccacgtctcaCACC-3′) and TRAP-oligo (BsmBI GGA)-R (5′-AGCACAAccgtcgtctccAAAC-3′). The PCR reaction was carried out using PrimeSTAR HS DNA Polymerase (Takara, Japan) following the manufacturer’s instructions. The PCR products of library oligos were then used for Golden Gate Assembly (GGA) to generate the plasmids library and the ligation products were transformed to chemically-induced competent DH5α cells. About 10 µl GGA ligation product was transformed, for each reaction, into 50 µl competent cells. All transformed cells were plated on one LB plate (∅15 cm) with Xgal, IPTG, and Amp selection. For one library containing all the synthetic oligos, 12 parallel transformations were performed, and all the bacterial colonies were scraped off and pooled together for plasmids midi-prep (PureLink™ HiPure Plasmid DNA Midiprep Kit).

### Lentivirus production and lentivirus titre quantification

The lentivirus was produced in HEK293T cells by co-transfection with packaging plasmids. Briefly, for lentivirus production in 10 cm dish, the DNA/PEI (Polyethylenimine Linear, MW 40000) mixture contains 13 µg pLenti-TRAPseq vectors, 3 µg pRSV-REV, 3.75 µg pMD.2 G, 13 µg pMDGP-Lg/p-RRE, 100 µg PEI 40000 solution (1 µg/µl in sterilised ddH2O) and supplemented by opti-MEM without phenol red (Invitrogen) to a final volume of 1 mL. The transfection mixture was pipetted up and down several times gently, and further incubated for keeping at room temperature (RT) for 20 min. The transfection complex was added to 80%-confluent HEK293T cells in a 10-cm dish containing 10 ml of culture medium. After 48 h viral supernatant was harvested, filtered through a 0.45 µm filter, and polybrene solution (Sigma-Aldrich) was added to the crude virus to a final concentration of 8 µg/mL. The crude virus was aliquoted into 15 mL tubes (5 mL/tube) and stored in a −80 °C freezer. Viral titre was estimated by counting the number of GFP-positive cells in the virus-treated population by flow cytometry (FCM) as follows: (1) HEK293T cells were split and seeded to a 24-well plate on day 1. Generally, gradient volumes of 5, 10, 20, 40, 80, 160 and 320 µl of crude lentivirus were added to the cells, and each volume was assayed in duplicate (Supplementary Fig. 18); (2) On day 2, lentivirus transduction was conducted when cells reached up to 60–80% confluence. Before transduction, the last two wells of cells were detached using 0.05% EDTA-Trypsin to determine the total number of cells in one well (*N*_initial_). Then the gradient volume of the crude virus was added to each well and swirled gently; (3) On day 3, we changed to a fresh medium; (4) On day 4, cells were harvested and washed twice in PBS. The cells were fixed in 4% formalin solution at RT for 20 min, then washed with PBS twice. FCM was performed using a BD LSRFortessa™ cell analyzer and the FACSDiva v.9.0 software with at least 50,000 events collected for each sample in replicates. The FCM output data was analysed with the FCM analysis software NovoExpress v.1.5.6. The percentage of GFP-positive cells, for all samples, was calculated as:

1 $$\begin{array}{c}Y \% =\frac{{{{{{\rm{Num}}}}}}.{{{{{\rm{GFP}}}}}}-{{{{{\rm{positive}}}}\; {{{\rm{cells}}}}}}}{{{{{{\rm{Num}}}}}}.{{{{{\rm{total}}}}\; {{{\rm{cells}}}}}}} \times {100}\end{array}$$

For accurate titre determination, there should be a linear relationship between the GFP-positive percentages and crude volume. The titre (transducing units (TU/mL)) was calculated according to the following formula in which *V* represents the crude volume (µl) used for initial transduction:

2 $$\begin{array}{c}\frac{{{{{{{\mathrm{TU}}}}}}}}{{{{{{{\mathrm{mL}}}}}}}}=\frac{{N{{_{{{\mathrm{initial}}}}}}} \times {Y \%} \times {1000}}{V}\end{array}$$

### Lentivirus library transduction

HEK293T cells stably expressing a low level of SpCas9 were infected at a MOI=0.3 to ensure that most cells receive only one viral construct with high probability. Overexpression of SpCas9 in the HEK293T cell line is induced by doxycycline (Dox). At day 1 (24 h after transduction), transduced cells in each dish were split into two dishes equally. On day 2 (48 h after transduction), the sub-group 1 was changed to a fresh medium containing 50 µg/ml hygromycin + 1 µg/mL puromycin (Dox-free group, or Dox-). The sub-group 2, instead, was changed to medium containing 50 µg/ml hygromycin + 1 µg/mL puromycin + 1 µg/mL doxycycline (Dox-induction group, or Dox+). The transduced cells were split every 2–3 days when cell confluence reaches up to 90%. After 6 and 10 days of selection, cells were harvested for genomic DNA extraction. Parallel experiments were performed using wild-type HEK293T cells.

### PCR amplicons of a surrogate library from cells

The genomic DNA was extracted with the phenol-chloroform method. To remove RNA contamination, the genomic DNA was digested with RNase A (OMEGA). Then the genomic DNA was subjected to PCR using PrimeSTAR polymerase (Takara, R045Q). The PCR primers were TRAP-NGS-F (5′-GGACTATCATATGCTTACCGTA-3′) and TRAP-NGS-R1 (5′-ACTCCTTTCAAGACCTAGCTAG-3′). The PCR products were purified by 1.5% gel and mixed with equal amounts and deep sequenced. The amplicons were subjected to deep sequencing on the MGISEQ-2000 (MGI of BGI in China) platform. All the samples were subjected to pair-ended 150 bp deep-sequencing.

### Data pre-processing

Raw sequencing reads were processed to obtain read counts (indel or total) for each surrogate target following our previously published method^17^. Briefly, the method consists of the following passages: quality assessment and cleaning of raw reads with FastQC v.0.11.3 (https://github.com/s-andrews/FastQC) and fastp v.0.19.6^44^; paired reads merge with FLASH v.1.2.1^45^; alignment to the reference library with BWA-MEM v.0.7.17^46^ (http://bio-bwa.sourceforge.net/); and reads quantification by extracting the aligned reads and identifying variations in the expected mapping pattern using pysam v.0.15.0^47^ (https://github.com/pysam-developers/pysam). For each target site, g + gRNA (20 bp) + scaffold (82 bp) + barcode (10 bp) + GTTT (terminal sequence) are guaranteed to remain unchanged when extracting reads at each random PAM site. To distinguish the variable PAM sites efficiently, we exploited the 10 bp barcode sequence at the beginning of each surrogate target sequence. Relative indel frequencies, expressed in percentage, were obtained separately from the read counts measured on day 6 and day 10 (Dox− or Dox+) respectively as:

3 $$\begin{array}{c}{{{{{{\mathrm{indel}}}}}}}\,{{{{{{\mathrm{frequency}}}}}}}\,\left( \% \right)=\frac{{{{{{{\mathrm{Num}}}}}}}.\,{{{{{{\mathrm{reads}}}}}}}\,{{{{{{\mathrm{with}}}}}}}\,{{{{{{\mathrm{indels}}}}}}}}{{{{{{{\mathrm{Num}}}}}}}.\,{{{{{{\mathrm{total}}}}}}}\,{{{{{{\mathrm{reads}}}}}}}}\times \,100\end{array}$$

The drop-out of our protocol was minimal, and we obtained the following total unique PAM contexts: gRNA for *BMP3* *n* = 251; *ADRA2C* *n* = 255; *CHRNB2* *n* = 250; *PAX5* *n* = 255. A threshold on the minimum number of total reads was defined by iteratively removing samples with less than *n* total reads (*n* from 0 to 200, step size of 5). The value of *n* with the lowest associated Spearman’s correlation *p* value between the indel frequencies measured on day 6 and day 10 was selected. This procedure was performed separately for Dox− and Dox+, for which the resulting minimum reads thresholds were 90 and 35, respectively. Indel frequencies from day 6 and day 10 were then averaged. This data are available as Supplementary Data 1. For each gRNA, multiple targets of one selected context were evaluated using different barcodes. These presented a mean normalised standard deviation of 0.03 and 0.11 in Dox+ and Dox−, and their efficiencies were averaged (Supplementary Fig. 9).

### Collection and pre-processing of external datasets

#### Merged Xiang et al. dataset (2021)

The dataset employed to study the relationship between binding free energy changes and on-target cleavage efficiency was obtained by merging the data of Kim et al.^14^ and Xiang et al.^17^ as previously described in Xiang et al.^17^. This dataset consists of 23,902 gRNA sequences and corresponding Cas9 efficiencies, measured as indel frequencies, at targets flanked by 5′-N_−1_GGN_+1_-3′ PAMs. The specificity of gRNAs (CRISPRspec) was evaluated with the CRISPRoff pipeline v.1.1.2^20^ on the human genome hg38. The following two gRNAs failed to be evaluated by the pipeline within 2 weeks due to the extreme presence of off-targets and were thus excluded: TAAAAATACAAAAAATTAGC, T(x20). We also removed 806 gRNAs with no match to the genome hg38 (randomised gRNAs). Next, the dataset was filtered to remove gRNAs likely to form sub-optimal structures with the scaffold. For this, pairwise binding probabilities of the bases in the sgRNAs (gRNAs + scaffold) were evaluated with RNAfold v.2.2.5^48^. The sgRNAs were compared to the optimal secondary structure of the scaffold, computed by solely folding the scaffold with RNAfold. This structure includes the crRNA:tracrRNA fusion loop and three tail loops^49^. The comparison consisted in measuring the Euclidean distance between binding probabilities for pairs of bases that bind the optimal structure, or the probability to be unbound for those bases that do not pair with any other in the optimal structure (1 – probability of pairing with any other base). For each of the two distance measures, we tested if the 5% sgRNAs with the highest distance to the optimal structure is less efficient than other sgRNAs (Mann–Whitney one-sided test, see Supplementary Fig. 2). A significant result was obtained for the unpaired bases (*p* = 3.68E-15) while for paired bases the distance was not highly pronounced (*p* = 5.31E-02). Hence, we removed from the dataset 1155 gRNAs with a high distance to the optimal structure in terms of the probability of bases unpaired in the optimal structure to not pairing with any other base in folded sgRNAs. Additionally, we filtered out gRNAs with indel frequencies at target sites <2% (*n* = 539). The resulting 21,402 gRNAs were split into a training set (*n* = 14,981) and a test set (*n* = 6421), keeping sequences with <4 differences in the 30mer (gRNA + target context) in the same subset. The analysis of the free energy change properties was carried out on the training set only, filtered down to 11,602 unique elements by removing gRNAs with low specificity (CRISPRspec <5). This last filter was not applied for the evaluation of the impact of local off-targets on gRNA specificity and efficiency.

#### Dataset by Lin et al. (2014)

The data from Lin et al. consists in 4 gRNAs: R-01, R-30, R-08 and R-25. DNA bulges were generated at any position on the targets by removal of single bases in the gRNAs. Sequences and efficiencies were retrieved from the corresponding publication^29^.

#### Dataset by Tsai et al. (2015)

From the GUIDE-seq dataset^30^, we analysed four gRNAs with at least one off-target cleaved more efficiently than the on-target. These were processed to remove off-targets with differences to the on-target PAM (3 nt NGG), or mismatches to the gRNA in the PAM-proximal 4 nt, or more than three total mismatches to the gRNA.

#### Dataset by Hart et al. (2015)

The dataset of Hart et al.^50^ (4239 gRNAs) was retrieved from the study of Haeussler et al. as dataset Hct1162lib1Avg^51^. In addition to the removal of one gRNA with no match in hg38, gRNAs not overlapping their target gene in the CDS annotations of GENCODE v.32^52^ were eliminated, resulting in 4184 gRNAs. The dataset was also filtered to remove gRNAs with high Euclidean distance to the optimal scaffold structure in terms of unpaired bases, using the same threshold identified for the top 5% distant gRNAs described above (Euclidean distance threshold = 3.41). The processed dataset comprises 4066 gRNAs. The efficiencies, measured as fold-change in gRNA abundance, were ranked-normalised with SciPy rankdata^53^.

#### Datasets by Kim et al. (2020)

The dataset of Cas9 variants activity and PAM compatibility of 30 gRNAs was downloaded from Kim et al.^16^. All targets were designed to be followed by the same context (TA) after 4-nt PAMs variated in all possible ways (NNNNTA, *n* = 4^4^ = 256 contexts), except for 15 AGGVB targets (V ∈ {A, C, G} and B ∈ {C, G, T}), which we excluded. We analysed Cas9 variants compatible with NGNN PAMs and all high-fidelity Cas9 variants except for evoCas9, which showed low mean indel frequency at targets with GG PAM binding site (mean =12.66%). The following number of gRNAs for each Cas9 variant were employed: SpCas9-HF1 *n* = 7400, Sniper-Cas9 *n* = 7395, HypaCas9 *n* = 7392, eSpCas9(1.1) *n* = 7388, wild-type SpCas9 *n* = 7382, SpCas9-NG *n* = 7379, xCas9 *n* = 7367.

### Thermodynamic properties of gRNAs and DNA targets

The gRNA folding minimum free energy change (Δ*G*_*U*_) was computed with RNAfold v.2.2.5^48^ using default options. Hybridisation free energy changes of complementary gRNA-target-DNA (Δ*G*_*H*_) and DNA–DNA interactions (Δ*G*_*O*_) were computed with the CRISPRoff pipeline v.1.1.2^20^. The pipeline also provides gRNA specificity information (CRISPRspec) if a list of targets in the genome is provided. The off-targets provided to CRISPRoff were searched in the human genome hg38 with RIsearch2 (v.2.1)^54^ with the options recommended for usage in combination with CRISPRoff: gRNA seed region from position 1 to 20, the maximum number of mismatches allowed = 6 with no constraint on a minimum number of consecutive matches in the seed, energy upper threshold = 10,000, maximum extension length on the seed = 0, output format = p3. The surrogate DNA targets used in the datasets of Kim et al. (2019–2020), Xiang et al.^17^, and in our validation datasets, that represent the surrogate duplicates of on-target sites and have unknown genomic positions, were not included in the search. CRISPRoff was executed with default parameters. Sub-optimal gRNA–DNA interactions were evaluated with an extended version of RIsearch v.1.1^55^ developed for this project (RIsearch v.1.2), to allow the scoring of sub-optimal gRNA–DNA interactions flanking sliding PAMs with positional weights on stacking base pairs (see https://rth.dk/resources/risearch). Given a gRNA as query and a DNA sequence as a target, RIsearch v.1.2 was executed with the following options: matrix of RNA–DNA nearest neighbour parameters 'Su95' (same as in CRISPRoff); force-start, to require the interaction to start at the 3′ end of the target; -w CRISPR_20nt_5p_3p, where 'CRISPR_20nt_5p_3p' is the name of the precompiled file containing the array of 19 weights for stacking base-pair contributions defined in CRISPRoff^20^. All gRNA–DNA interactions were forced to end at the PAM. The optimal start position was searched by iteratively shortening the DNA target sequence by 1 nt from an initial length of 24 nt upstream of the PAM to a minimum of 2 nt. For targets at PAMs overlapping while sliding 1 nt up/downstream only up to 21 nt were effectively utilised by the algorithm to find optimal gRNA–DNA interactions. This number varies in the case of the data from Lin et al. and GUIDE-seq. The DNA–DNA opening free energy change at bindings with bulges or mismatches was calculated for the sequence stretch involved in the gRNA–DNA interaction, which may be longer or shorter compared to the fully complementary case. For this, the function for DNA–DNA binding free energy change computation of CRISPRoff was used. In the data from Lin et al. and GUIDE-seq, in which gRNA–DNA bindings can have different lengths, out of all possible sub-optimal interactions the one with the lowest ∆*G*_*B*_ was considered the optimal one. For all RNA–RNA, RNA–DNA and DNA–DNA stacking interactions, free energy change parameters were obtained from RIsearch2, which includes parameters for DNA–DNA^56,57^, RNA–RNA^58^ and RNA–DNA^59–61^ stacking interactions, as previously described^20^. Missing parameters, such as those for stacked bulges in RNA–DNA interactions, were obtained by averaging the corresponding RNA–RNA and DNA–DNA parameters, as in previous studies^20,62^.

### Integration of local sliding PAMs in CRISPRspec

Local sliding PAMs were included in the CRISPRspec score measuring binding competition by considering bindings flanking sliding PAMs as pseudo-off-targets and by including them in the partition function calculation of CRISPRspec. By linearly fitting to the efficiency, we estimate the weighted contributions of bindings attributed to the local sliding PAMs and added it on top of the original CRISPRspec measure obtained from the CRISPRoff pipeline v.1.1.2^20^. Off-targets with up to six mismatches in the human genome hg38 were searched with RIsearch2 (v.2.1)^54^ as explained above. Given the set *S* of all gRNAs, let *S*_up_ and *S*_down_ be the subsets of gRNAs in *S* that have respectively upstream and downstream local sliding PAMs. For a gRNA *x*, let *M* be the median function, |z| the absolute value of z, $\triangle {G}_{B}\left[{x}_{{up}}\right]$ and $\triangle {G}_{B}\left[{x}_{{down}}\right]$ the ∆*G*_*B*_ free energy changes computed at up- and down-stream binding sites of *x*, and *y*[*x*] the experimentally measured efficiency of *x*. Then, the extended CRISPRspec, CRISPRspecExt, of gRNA *x* takes the local sliding PAMs into account as follows:

4 $${{{{{\rm{CRISPRspecExt}}}}}}\left[x\right]=	\,{\alpha }_{0}+{\alpha }_{1}{{{{{\rm{CRISPRspec}}}}}}\left[x\right]\\ 	+{\lambda }_{{{{{{{\mathrm{up}}}}}}}}\left[x\right]\left({\beta }_{0}+{\beta }_{1}\left|\triangle {G}_{B}\left[{x}_{{{{{{{\mathrm{up}}}}}}}}\right]-M\left(\left\{\triangle {G}_{B}\left[i\right],\,\forall \,i\in {S}_{{{{{{{\mathrm{up}}}}}}}}\right\}\right)\right|\right)\\ 	+{\lambda }_{{{{{{{\mathrm{down}}}}}}}}\left[x\right]\left({\gamma }_{0}+{\gamma }_{1}\left|\triangle {G}_{B}\left[{x}_{{{{{{{\mathrm{down}}}}}}}}\right]-M\left(\left\{\triangle {G}_{B}\left[i\right],\,\forall \,i\in {S}_{{{{{{{\mathrm{down}}}}}}}}\right\}\right)\right|\right)$$

With:

5 $${\lambda }_{{{{{{{\mathrm{up}}}}}}}}\left[x\right]=\,\left\{\,\begin{array}{cc}0&{if}\,x\;\notin\; {S}_{{{{{{{\mathrm{up}}}}}}}}\hfill\\ 1& {if}\,x\in {S}_{{{{{{{\mathrm{up}}}}}}}}\end{array}\right.$$

6 $${\lambda }_{{{{{{{\mathrm{down}}}}}}}}\left[x\right]=\,\left\{\,\begin{array}{cc}0&{if}\,x\;\notin\; {S}_{{{{{{{\mathrm{down}}}}}}}}\hfill\\ 1&{if}\,x\in {S}_{{{{{{{\mathrm{down}}}}}}}}\end{array}\right.$$

The optimal least squares coefficients ${\hat{\alpha }}_{0}$, ${\hat{\alpha }}_{1}$, ${\hat{\beta }}_{0}$, ${\hat{\beta }}_{1}$, ${\hat{\gamma }}_{0}$, ${\hat{\gamma }}_{1}$ were estimated as:

7 $$\begin{array}{c}{\hat{\alpha }}_{0},{\hat{\alpha }}_{1}={{{{{{\rm{argmin}}}}}}}_{{{{{{{\rm{\alpha }}}}}}}_{0},{\alpha }_{1}}\mathop{\sum}\limits _{x\in S}{\left(y\left[x\right]-{\alpha }_{0}-{\alpha }_{1}{CRISPRspec}\left[x\right]\right)}^{2}\end{array}$$

8 $$\begin{array}{c}{\hat{\beta }}_{0},{\hat{\beta }}_{1}={{{{{{\rm{argmin}}}}}}}_{{{{{{{\rm{\beta }}}}}}}_{0},{\beta }_{1}}\!\mathop{\sum}\limits_{x\in {S}_{{{{{{{\mathrm{up}}}}}}}}}{\left(y\left[x\right]\!-\!{\beta }_{0}-{\beta }_{1}\left|\triangle {G}_{B}\left[{x}_{{{{{{{\mathrm{up}}}}}}}}\right]\!-\!M\left(\left\{\triangle {G}_{B}\left[i\right],\forall i\;\in\; {S}_{{{{{{{\mathrm{up}}}}}}}}\right\}\right)\right|\right)}^{2}\end{array}$$

9 $${\hat{\gamma }}_{0},{\hat{\gamma }}_{1}\!=\!{{{{{{\rm{argmin}}}}}}}_{{{{{{{\rm{\gamma }}}}}}}_{0},{\gamma }_{1}}\!\mathop{\sum}\limits_{x\in {S}_{{{{{{{\mathrm{down}}}}}}}}}{\left(y\left[x\right]-{\gamma }_{0}\!-\!{\gamma }_{1}\left|\triangle {G}_{B}\left[{x}_{{{{{{{\mathrm{down}}}}}}}}\right]\!-\!M\left(\left\{\triangle {G}_{B}\left[i\right],\forall i\;\in\; {S}_{{{{{{{\mathrm{down}}}}}}}}\right\}\right)\right|\right)}^{2}$$

Hence, parameters for local sliding PAMs are estimated from the absolute deviation of Δ*G*_*B*_ to the median Δ*G*_*B*_ of all local sliding PAMs of the same type (up/down) in the training set.

### Statistical analysis

The SciPy v.1.5.0-1.6.3^53^, NumPy v.1.18.5^63^, pandas v.1.2.0^64^ and scikit-learn v.0.23.1^65^ modules were applied for data analysis in Python v.3.8.3^66^. Nucleotide strings were managed in Biopython v.1.77. Plots were generated using Matplotlib v.3.2.2 and seaborn v.0.11.1. The impact of gRNA free energy change properties in defining gene knockout efficiency was analysed after sorting and separating gRNAs into two groups, high (top 20%) and low (bottom 20%) efficient, based on their reported indel frequencies (Supplementary Fig. 19). The intervals of preferential values identified for each free energy change property were set to contain 80% of the total highly efficient gRNAs. The portion of efficiency variance associated to the differences in the context of the 'GG' PAM binding site that could be explained by the sliding model was computed as the sum of squares (SSQ_sliding_) between the efficiencies of each sliding condition and the grand mean (GM) divided by the total sum of squares between the efficiencies and the grand mean (SSQ_tot_):

10 $$\begin{array}{c}{{{{{\rm{Explained}}}}}}\; {{{{{\rm{variance}}}}}}=\,\frac{{{{{{{{\mathrm{SSQ}}}}}}}}_{{{{{{{\mathrm{sliding}}}}}}}}}{{{{{{{{\mathrm{SSQ}}}}}}}}_{{{{{{{\mathrm{tot}}}}}}}}}\end{array}$$

With:

11 $$\begin{array}{c}{{{{{{{\mathrm{SSQ}}}}}}}}_{{{{{{{\mathrm{sliding}}}}}}}}={n}_{{{{{{{\mathrm{up}}}}}}}}{\left({M}_{{{{{{{\mathrm{up}}}}}}}}-{{{{{{\mathrm{GM}}}}}}}\right)}^{2}+{n}_{{{{{{{\mathrm{down}}}}}}}}{\left({M}_{{{{{{{\mathrm{down}}}}}}}}-{{{{{{\mathrm{GM}}}}}}}\right)}^{2}+\\ +\,{n}_{{{{{{{\mathrm{up}}}}}}}{{\& }}{{{{{{\mathrm{down}}}}}}}}{\left({M}_{{{{{{{\mathrm{up}}}}}}}{{\& }}{{{{{{\mathrm{down}}}}}}}}-{{{{{{\mathrm{GM}}}}}}}\right)}^{2}+{n}_{{{{{{{\mathrm{none}}}}}}}}{\left({M}_{{{{{{{\mathrm{none}}}}}}}}-{{{{{{\mathrm{GM}}}}}}}\right)}^{2}\end{array}$$

12 $$\begin{array}{c}{{{{{{{\mathrm{SSQ}}}}}}}}_{{{{{{{\mathrm{tot}}}}}}}}=\mathop{\sum}\limits _{s\in S}{\left({x}_{s}-{{{{{{\mathrm{GM}}}}}}}\right)}^{2}\end{array}$$

13 $$\begin{array}{c}{{{{{{\mathrm{GM}}}}}}}=\,\frac{{n}_{{{{{{{\mathrm{up}}}}}}}}{M}_{{{{{{{\mathrm{up}}}}}}}}+\,{n}_{{{{{{{\mathrm{down}}}}}}}}{M}_{{{{{{{\mathrm{down}}}}}}}}+\,{n}_{{{{{{{\mathrm{up}}}}}}}{{\& }}{{{{{{\mathrm{down}}}}}}}}{M}_{{{{{{{\mathrm{up}}}}}}}{{\& }}{{{{{{\mathrm{down}}}}}}}}+\,{n}_{{{{{{{\mathrm{none}}}}}}}}{M}_{{{{{{{\mathrm{none}}}}}}}}}{{n}_{{{{{{{\mathrm{up}}}}}}}}+\,{n}_{{{{{{{\mathrm{down}}}}}}}}+\,{n}_{{{{{{{\mathrm{up}}}}}}}{{\& }}{{{{{{\mathrm{down}}}}}}}}+\,{n}_{{{{{{{\mathrm{none}}}}}}}}}\end{array}$$

Where S is the set of all gRNAs with a 'GG' PAM binding site, *x*_*s*_ is the efficiency of a gRNA, and *n*_up_, *n*_down_, *n*_up&down_, *n*_none_, *M*_up_, *M*_down_, *M*_up&down_, *M*_none_ are the size (*n*) and the mean (*M*) of respectively the data subsets split by sliding categories: upstream (up); downstream (down); upstream and downstream (up&down); no sliding (none).

### Reporting Summary

Further information on research design is available in the Nature Research Reporting Summary linked to this article.

## Supplementary information

Supplementary Information

Description of Additional Supplementary Files

Supplementary Data 1

Supplementary Data 2

Reporting Summary

## Data Availability

The raw sequencing data generated in this study have been deposited in the NCBI Sequence Read Archive under accession code BioProject: PRJNA732236 and in the China National GeneBank under accession code CNP0001874. The primary sequence of the human genome hg38 was downloaded from the NCBI, RefSeq assembly accession GCF_000001405.38. Additional data used in this study are available as supplementary material in the following publications: dataset by Xiang et al.^17^ [10.1038/s41467-021-23576-0], dataset by Kim et al.^14^ [10.1126/sciadv.aax9249], dataset by Lin et al^29^. [10.1093/nar/gku402], dataset by Tsai et al.^30^ [10.1038/nbt.3117], dataset by Hart et al.^50,51^ [10.1186/s13059-016-1012-2], datasets by Kim et al.^16^ [10.1038/s41587-020-0537-9]. Source data for the figures and supplementary figures are provided as a Source Data file. Source data are provided with this paper.

## Source data

Source Data

## Code availability

The source code of RIsearch v.1.2 is available under the GNU General Public Licence v.3 via https://github.com/RTH-tools/risearch/ and https://rth.dk/resources/risearch/.
