# Supplementary material for: CRISPR/Cas9 gRNA activity depends on free energy changes and on the target PAM context
Source: Nat Commun. 2022 May 30;13:3006. doi: 10.1038/s41467-022-30515-0 (PMC9151727; doi:10.1038/s41467-022-30515-0)
Supplement: Supplementary file 1 — Supplementary Information [file 41467_2022_30515_MOESM1_ESM.pdf]

Supplementary information

CRISPR/Cas9 gRNA activity depends on free energy changes and on the target PAM context

Giulia I. Corsi[1*], Kunli Qu[2,3*], Ferhat Alkan[1,4], Xiaoguang Pan[2], Yonglun Luo[2,5,6,7‡] and Jan Gorodkin[1‡]

Affiliations

[1]Center for non-coding RNA in Technology and Health, Department of Veterinary and Animal Sciences, University of Copenhagen, Thorvaldsensvej 57, Frederiksberg 1871, Denmark.

[2]Lars Bolund Institute of Regenerative Medicine, Qingdao-Europe Advanced Institute for Life Sciences, BGI-Qingdao, Qingdao 266555, China.

[3]Department of Biology, University of Copenhagen, Copenhagen 2200, Denmark.

[4]Division of Oncogenomics, The Netherlands Cancer Institute, Plesmanlaan 121, 1066 CX Amsterdam, The Netherlands.

[5]BGI-Shenzhen, Shenzhen 518083, China.

[6]Department of Biomedicine, Aarhus University, Aarhus 8000, Denmark.

[7]Steno Diabetes Center Aarhus, Aarhus University Hospital, Aarhus 8200, Denmark.

[*]These authors contributed equally: Giulia I. Corsi, Kunli Qu

‡These authors jointly supervised this work: Yonglun Luo (experimental) and Jan Gorodkin (computational). Email: alun@biomed.au.dk, gorodkin@rth.dk

Table of Contents

Supplementary Figures 3

Supplementary Figure 1. Comparison between hybridization free energy change and GC content. 3

Supplementary Figure 2. Low accessibility of bases expected to be unbound in the sgRNA scaffold results in low efficiency 4

Supplementary Figure 3. Position-specific free energy change profile of gRNA bindings. 5

Supplementary Figure 4. Influence of thymine at the gRNA seed 3' end. 5

Supplementary Figure 5. Impact of multiple local sliding PAMs. 6

Supplementary Figure 6. Binding free energy change ΔGB at local sliding PAMs. 7

Supplementary Figure 7. CRISPRspecExt extending on CRISPRspec to include binding contributions from local sliding PAMs. 8

Supplementary Figure 8. Correlation between binding competition and efficiency. 9

Supplementary Figure 9. Filtering and processing of in-cell validation data. 10

Supplementary Figure 10. Correlation between gRNA efficiencies measured in Dox+ and Dox- cells. 11

Supplementary Figure 11. Indel frequency at PAM binding sites with various sequences in Dox+ HEK293T cells. 11

Supplementary Figure 12. Indel frequency at local sliding upstream PAMs in Dox- HEK293T cells. 12

Supplementary Figure 13. Indel frequency at local sliding downstream PAMs in Dox- HEK293T cells. 13

Supplementary Figure 14. Indel frequency at local sliding upstream PAMs in Dox+ HEK293T cells. 14

Supplementary Figure 15. Indel frequency at local sliding downstream PAMs in Dox+ HEK293T cells. 15

Supplementary Figure 16. Indel frequency at alternative PAM binding sites AG and GA with variable context in HEK293T cells. 16

Supplementary Figure 17. Indel frequency of wild-type SpCas9 at PAM binding sites with variated sequences, Kim et al. (2020) dataset. 17

Supplementary Figure 18. Quantification of lentiviral library titer. 17

Supplementary Figure 19. Partition of gRNAs in efficiency groups. 18

Supplementary Tables 18

Supplementary Table 1. Dataset pre-processing and filtering. 18

Supplementary Table 2. Contingency table of hybridization free energy change. 19

Supplementary Table 3. Contingency table of DNA-DNA binding free energy change. 19

Supplementary Table 4. Contingency table of minimum gRNA self-folding free energy change. 20

Supplementary Table 5. Contingency table of ΔGB residual binding free energy change. 20

Supplementary Table 6. Stacking base pair free energy change contributions. 20

Supplementary Table 7. Free energy change properties of gRNAs forming DNA-bulged gRNA-DNA interactions. 21

Supplementary Table 8. gRNA sequences selected to evaluate Cas9-mediated cleavage efficiency at surrogate target sites with different PAMs. 22

References 22

Supplementary Figures

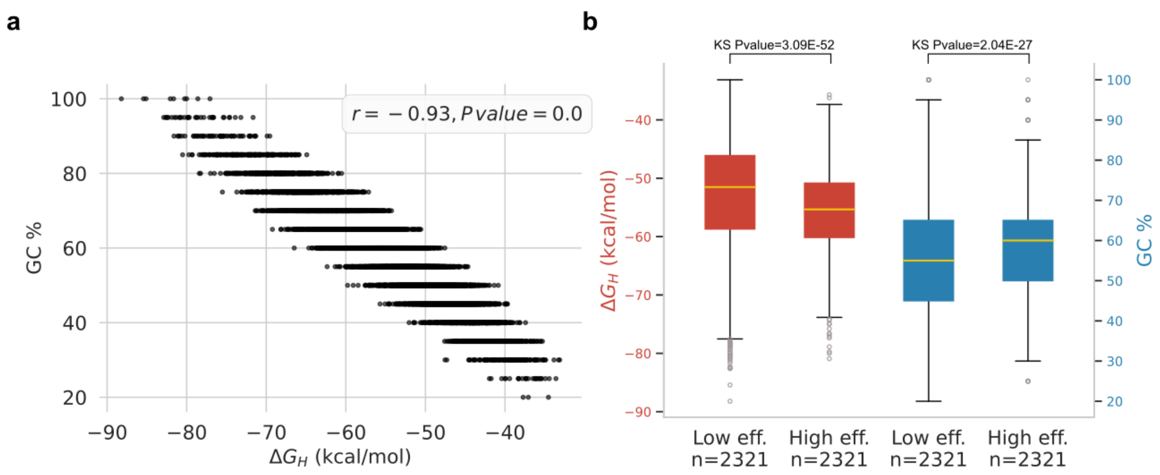

Supplementary Figure 1. Comparison between hybridization free energy change and GC content.

(**a**) Correlation between the GC content (Y-axis) and the ΔG_H hybridization free energy change (X-axis). The Pearson's r and P-value (two-sided, not truncated) are indicated in the plot. (**b**) Box plots describing the GC content (Y-axis right) and the ΔG_H (Y-axis left) of efficient and inefficient gRNAs in the dataset. Statistical significance is computed via the two-sample Kolmogorov-Smirnov test, two-sided. Although the test is more conservative for the GC%, which is discrete for sequences of fixed length, the difference between the two properties is substantial. Boxes represent the 1st and 3rd quartiles (Q1, Q3), with the median represented by a line; whiskers extend up (or down) to the last (or first) value lower (or greater) than Q3+1.5*(Q3-Q1) (or Q1-1.5*(Q3-Q1)).

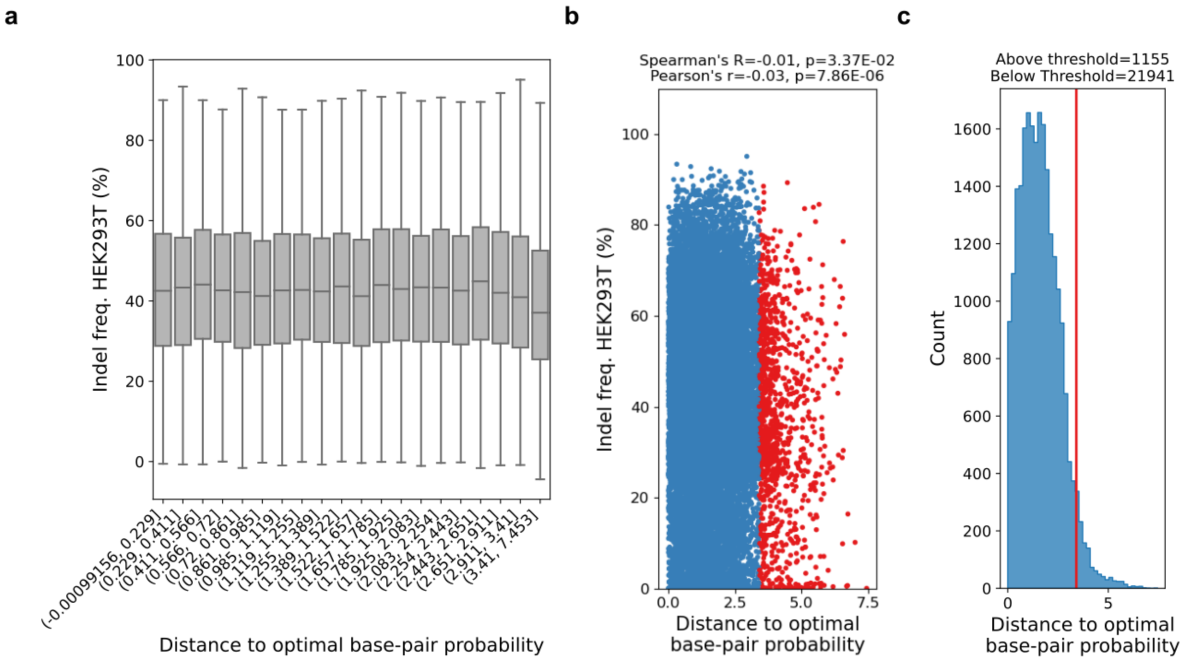

Supplementary Figure 2. Low accessibility of bases expected to be unbound in the sgRNA scaffold results in low efficiency.

(**a**) Boxplot showing the indel frequency of gRNAs in merged Xiang et al. (2021) dataset, filtered by removing gRNAs with no match in hg38 (*n*=23,096), split in percentiles of 5% based on the Euclidean distance to the optimal accessibility probabilities of bases expected to be unbound in the secondary structure of the sgRNA scaffold. Boxes are defined as in **Figure S1**. (**b**) Scatter plot of the data in (**a**), with the 5% of the data with highest Euclidean distances coloured in red. Two-sided correlation tests are reported on top of the image. (**c**) Histogram of Euclidean defined as in (**a**). A red vertical bar indicates the value of percentile 95.

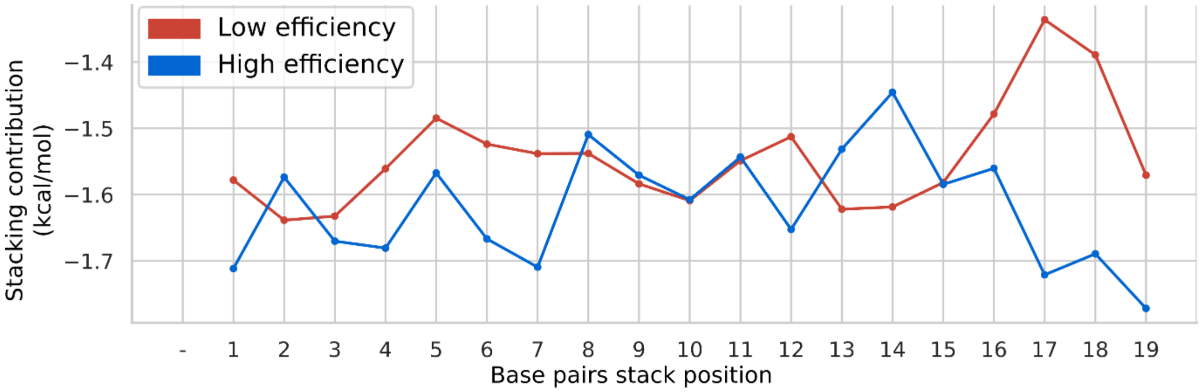

Supplementary Figure 3. Position-specific free energy change profile of gRNA bindings. Mean base pair stacking free energy changes at each position in the gRNA-DNA hybrid (not weighted).

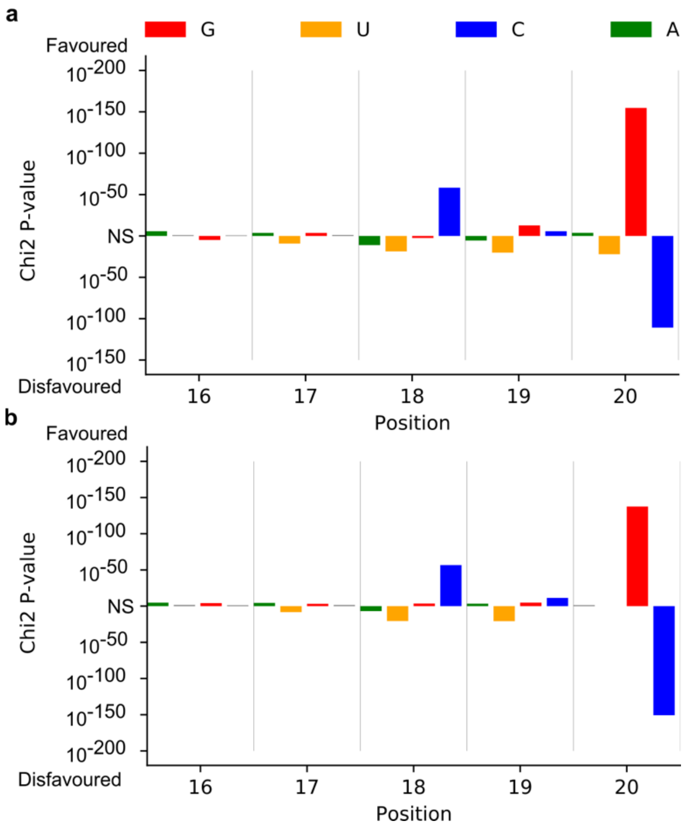

Supplementary Figure 4. Influence of thymine at the gRNA seed 3’ end. Position-specific nucleotides at the gRNA seed 3’ end favourable and unfavourable for cleavage (positions 16-20 in the gRNA). gRNAs with up to 2 Us in the last 5 nt of the seed 3’ end (**a**) and with no U at position 20 (**b**). The p-value relative to the Chi-squared contingency test (SciPy[1]) is reported on the Y-axis, split into “favoured” and “disfavoured” based on the modal efficiency class of gRNAs bearing the property.

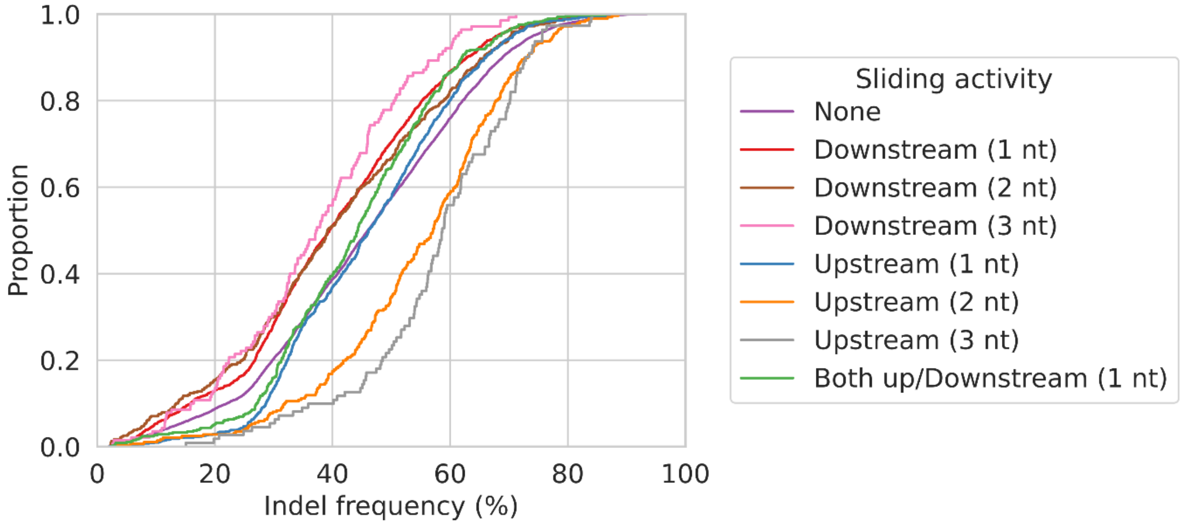

Supplementary Figure 5. Impact of multiple local sliding PAMs.

Cumulative distributions of indel frequencies of gRNAs grouped by the presence of local sliding PAMs at their targets. The distance between the on-target binding site and the local sliding PAM (interspaced by Gs) is stated in parenthesis. The groups are disjoint. Number of gRNAs Downstream (1, 2, 3 nt) n=1630, 469, 140; Upstream (1, 2, 3 nt) n=942, 286, 111; None n=7,565; Both up&downstream (1 nt) n=459. One-way ANOVA p-value between groups: Downstream (1, 2, 3 nt)=0.14; Upstream (1, 2, 3 nt)=8.39E-23.

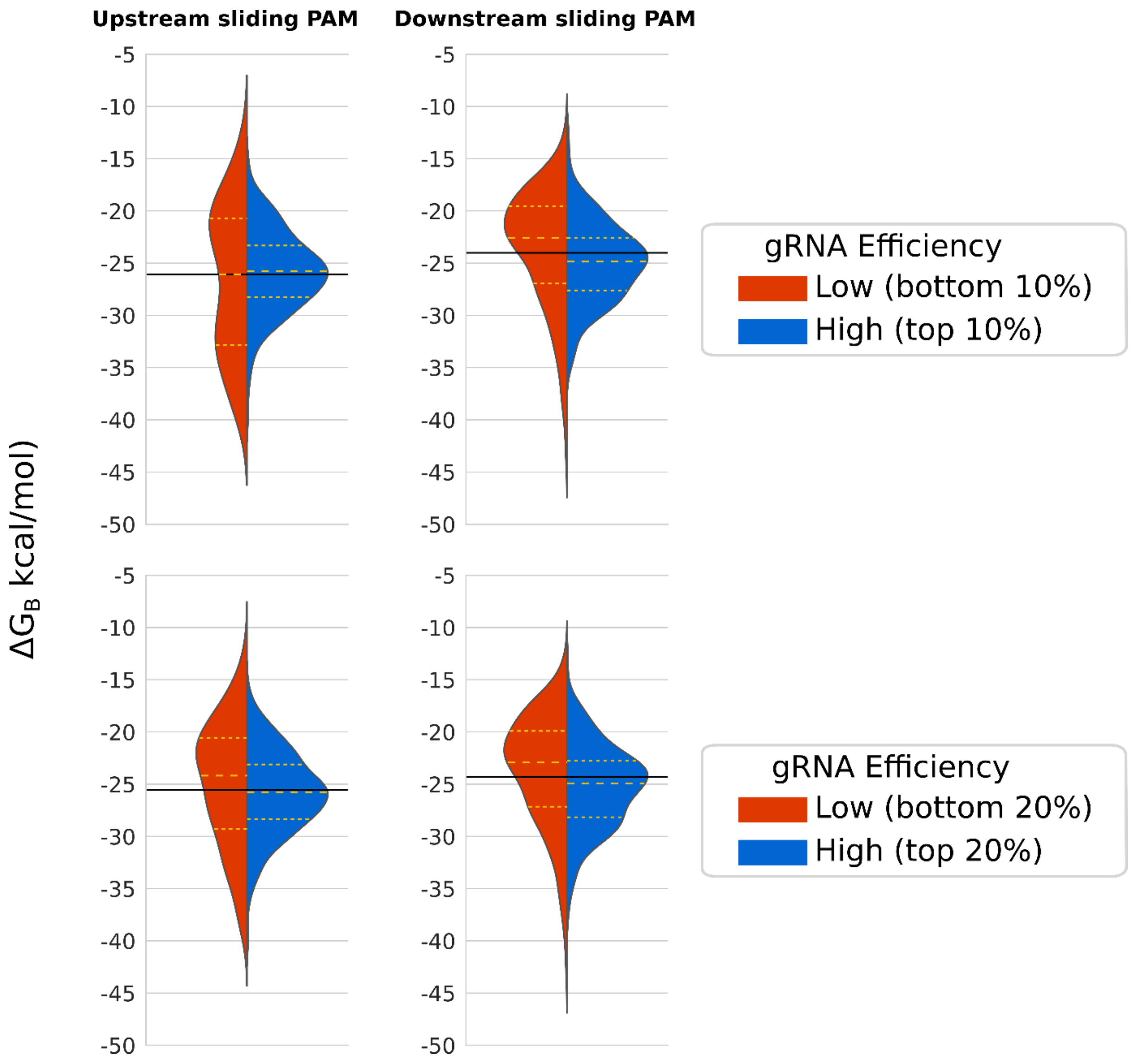

Supplementary Figure 6. Binding free energy change ΔG_B at local sliding PAMs.
Violin plots comparing the ΔG_B of highly efficient (top 10% or 20%) and low-efficient (bottom 10% or 20%) gRNAs binding at targets with an upstream (left) or downstream (right) local sliding PAM. Dashed lines represent the quartiles and medians of the groups. A black solid line indicates the median ΔG_B of a category (regardless of the efficiency). gRNAs that can bind at targets with both up- and down-stream local sliding PAMs are in both plots. Number of gRNAs split by efficiency (10% threshold) with local sliding PAMs at targets upstream: low=72, high=185; downstream: low=368, high=147; (20% threshold) upstream: low=215, high=382; downstream: low=691, high=338. Total gRNAs with local sliding PAMs at targets upstream=1798; downstream=2698.

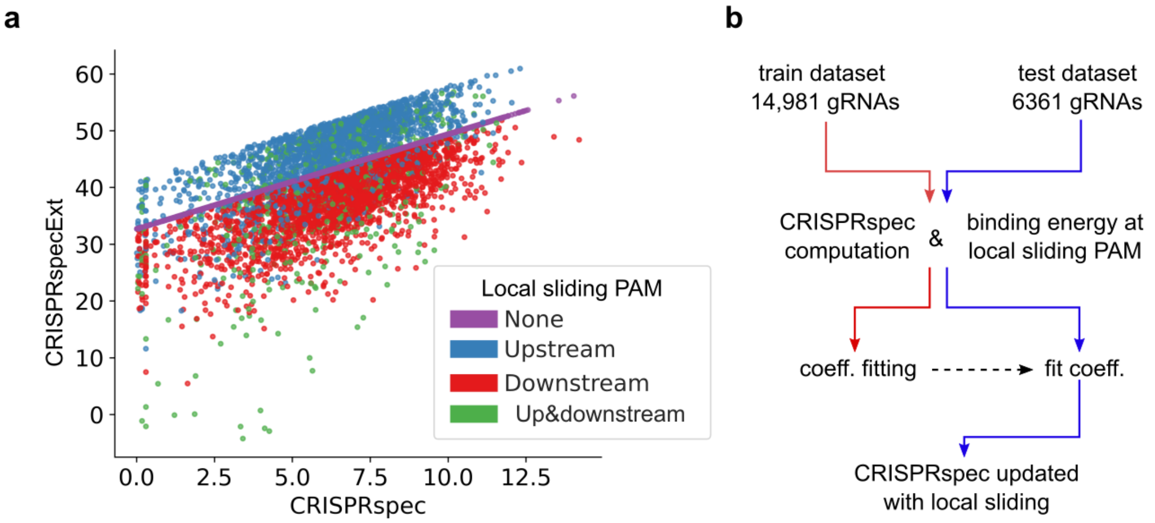

Supplementary Figure 7. CRISPRspecExt extending on CRISPRspec to include binding contributions from local sliding PAMs.

(**a**) The impact of local sliding PAMs on gRNA specificity is shown by comparing specificity scores without (CRISPRspec) and with (CRISPRspecExt) taking into account the local sliding PAMs. The colour of each dot reflects the presence/absence of different types of local sliding PAMs. The upper linear "bound" is due to the dominance of the estimated positive intercept (β_0=8.54) over the negative coefficient (β_1=-1.41) in the linear fitting applied to upstream local sliding PAMs. This creates an upper limit of positive specificity increase for gRNAs with binding free energy change ΔG_B close to the median of the group (see Methods for details on the linear model applied). The bound is not present in downstream local sliding PAMs, whose estimated intercept and coefficient have the same sign (γ_0=-0.04 and γ_1=-1.30). Note that for gRNAs with no local sliding PAM at their targets CRISPRspec is simply re-scaled (α_0=32.68, α_1=1.67). (**b**) Schematic representation of the pipeline used for parameter fitting and testing.

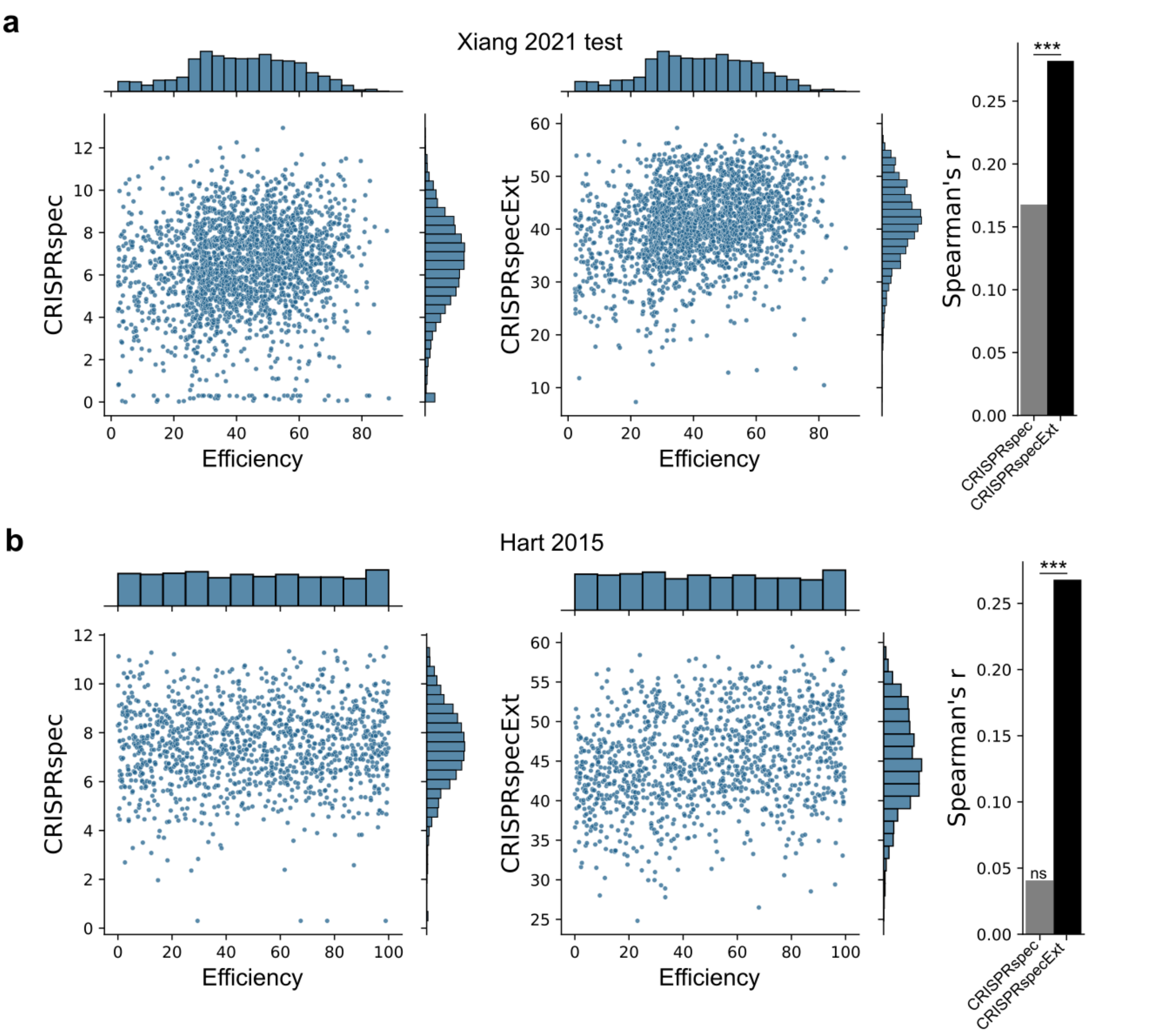

Supplementary Figure 8. Correlation between binding competition and efficiency.
The relation between gRNA efficiency and binding competition measured by scores accounting for global (CRISPRspec) and global plus local (CRISPRspecExt) off-targets is presented for two datasets: (**a**) The merged dataset “Xiang 2021 test”, n=2219 gRNAs; and (**b**) “Hart 2015”, n=1241 gRNAs. All comparisons present positive correlations, except for CRISPRspec in Hart 2015 (**b**, left), which is not significantly (ns) correlated to the efficiency. Spearman’s correlations and two-sided p-values: “Xiang 2021 test”, CRISPRspec R=0.17, p-value=1.81E-15 and CRISPRspecExt R=0.28, p-value=7.42E-42; “Hart 2015”, CRISPRspec R=0.04, p-value=0.15 and CRISPRspecExt R=0.27, p-value=7.72E-22. Of note, also the correlation between CRISPRspec and the indel frequency values reported in the portion of the merged Xiang 2021 dataset used for training is positive (Spearman’s R=0.18, p=1.97E-39). The significance of the improvement between CRISPRspec and CRISPRspecExt is calculated via the one-sided Steiger’s test p-value=1.85E-09 in “Xiang 2021 test” and p-value=4.44E-16 in “Hart 2015”. ***p<0.001.

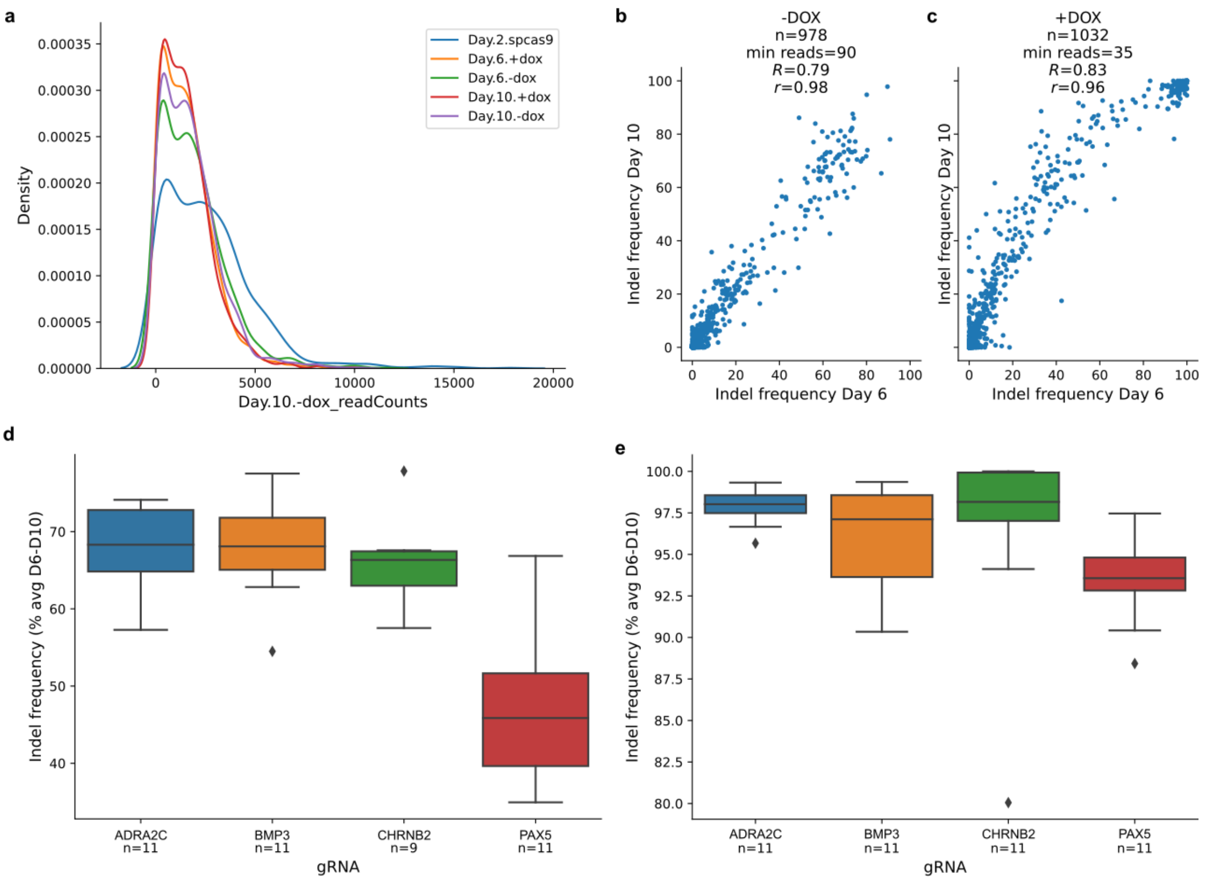

Supplementary Figure 9. Filtering and processing of in-cell validation data.

(**a**) Distribution of read counts obtained from deep sequencing in HEK293T cells untreated (Dox-) or treated (Dox+) measured 2, 6 and 10 days after transduction. (**b-c**) Spearman's *R* and Pearson's *r* correlation between indel frequencies at day 6 and day 10. The data was pre-filtered to remove gRNA targets with reads count < 90 in Dox- cells (**b**) or < 35 in Dox+ (**c**). (**d-e**) Indel frequencies of gRNA-target-DNA replicates for targets with $N_{-1}GGN_{+1}$ PAMs with sequences: AGGC (BMP3); AGGG (CHRNB2); AGGC (ADRA2C); TGGA (PAX5) in Dox- (**d**) and Dox+ (**e**) cells. The number of elements in each box is reported on the X-axis. Boxes are defined as in **Supplementary Fig. 1**.

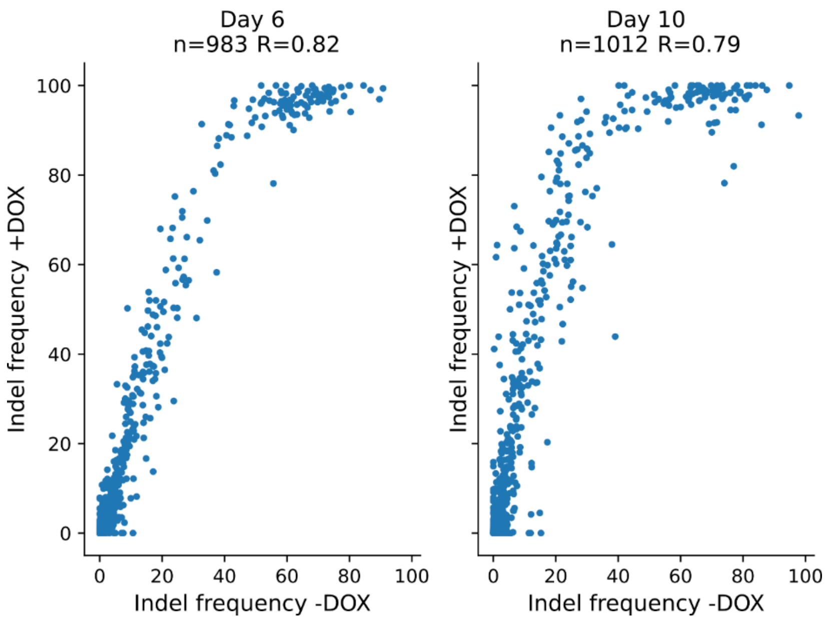

Supplementary Figure 10. Correlation between gRNA efficiencies measured in Dox+ and Dox- cells.

Indel frequencies measured in cells 6 and 10 days after transduction in Dox+ and Dox- HEK293T cells. The Spearman's correlation (R) is indicated.

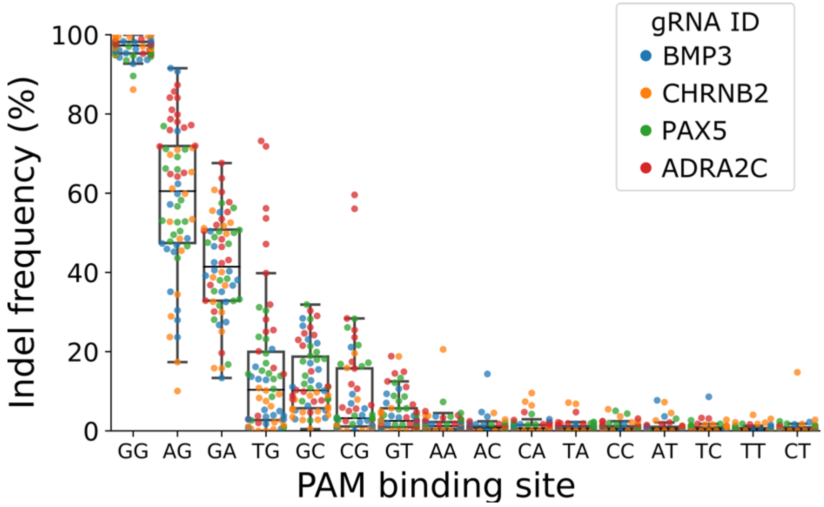

Supplementary Figure 11. Indel frequency at PAM binding sites with various sequences in Dox+ HEK293T cells.

Indel frequency of gRNAs binding at DNA targets with different PAM binding sites (X-axis), measured in cells treated with Doxycycline (Dox+). The swarm plot details the indel frequencies of each of 4 gRNA, for target DNA sites carrying different PAM contexts ($N_{-1}N_{+1}$ in $N_{-1}NNN_{+1}$ where the middle NN is the PAM binding site). Boxes are defined as in **Supplementary Fig. 1**. Number of gRNAs in each box, left to right: 63; 62; 60; 62; 62; 61; 63; 61; 61; 62; 61; 63; 62; 62; 64; 63.

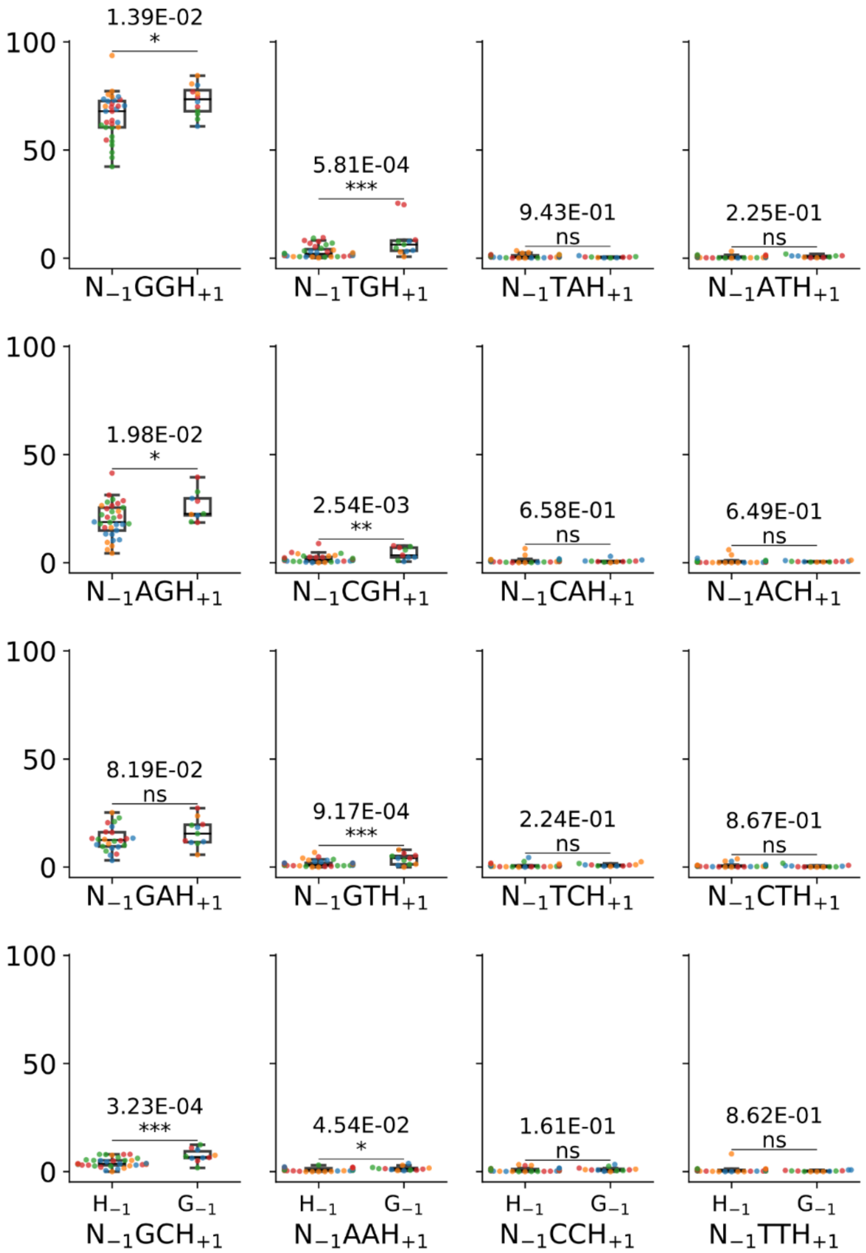

Supplementary Figure 12. Indel frequency at local sliding upstream PAMs in Dox-HEK293T cells.

Indel frequency at DNA targets with a G upstream from the PAM binding site (G_{-1}) compared to targets with no upstream G (H_{-1}). Binding sites contexts with $G_{+}1$ or with efficiencies for less than 3 of 4 gRNAs were excluded. The indel frequency was measured in non-treated cells (Dox-). The boxes and the swarm plot are defined as in **Supplementary Fig. 1 and 11**. One-sided t-test p-values are indicated on top of the boxes (the alternative hypothesis is that gRNA efficiencies at G_{-1} targets have larger mean than H_{-1} targets). Number of elements in each box, from top to bottom, left to right: 32; 12; 33; 9; 24; 11; 33; 10; 35; 11; 31; 9; 36; 11; 35; 12; 31; 19; 35; 11; 34; 11; 35; 12; 35; 10; 33; 12; 34; 12; 36; 12.

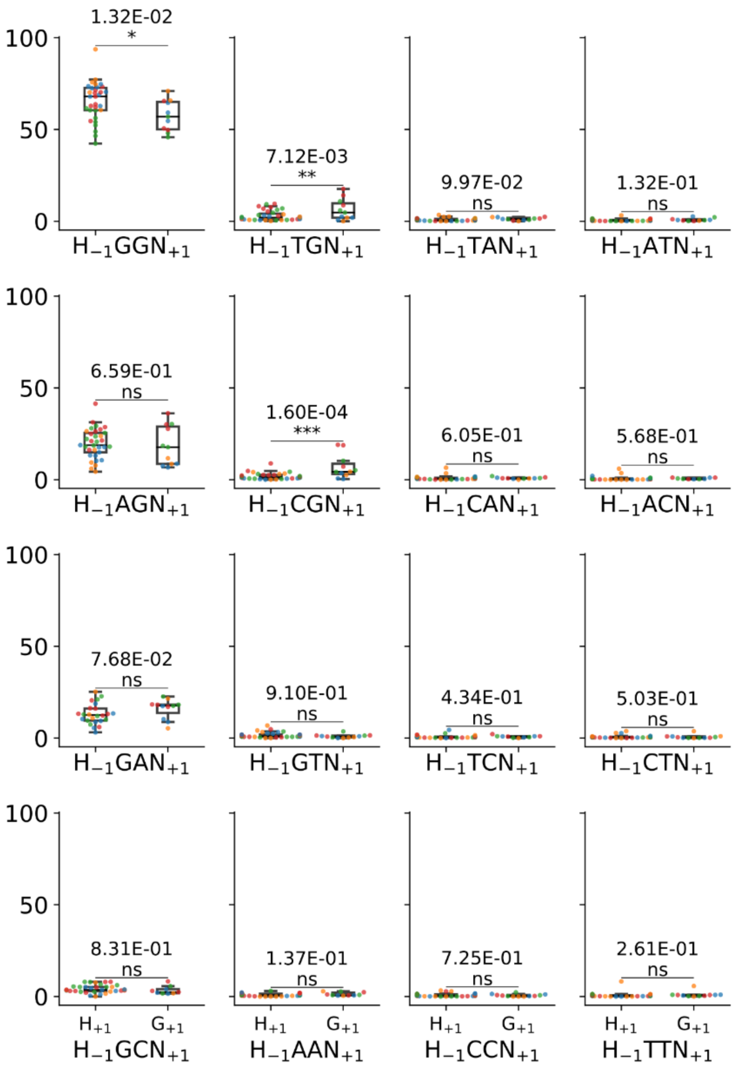

Supplementary Figure 13. Indel frequency at local sliding downstream PAMs in Dox- HEK293T cells.

Indel frequency at DNA targets with a G downstream from the PAM binding site (G_{+1}) compared to targets with no downstream G (H_{+1}). Binding sites contexts with G_{-1} or with efficiency for less than 3 of 4 gRNAs were excluded. The indel frequency was measured in non-treated cells (Dox-). The boxes and the swarm plot are defined as in **Supplementary Fig. 1 and 11**. One-sided t-test p-values are indicated on top of the boxes (the alternative hypothesis is that gRNA efficiencies at G_{+1} targets have larger mean than H_{+1} targets, except for the "GG" PAM binding site (top left) for which the alternative hypothesis is that the mean gRNA efficiency at G_{+1} targets is less than at H_{+1} targets). Number of elements in each box, from top to bottom, left to right: 31; 11; 33; 11; 24; 11; 33; 9; 35; 11; 31; 11; 36; 12; 35; 10; 31; 11; 35; 11; 34; 10; 35; 12; 35; 10; 33; 9; 34; 12; 36; 11.

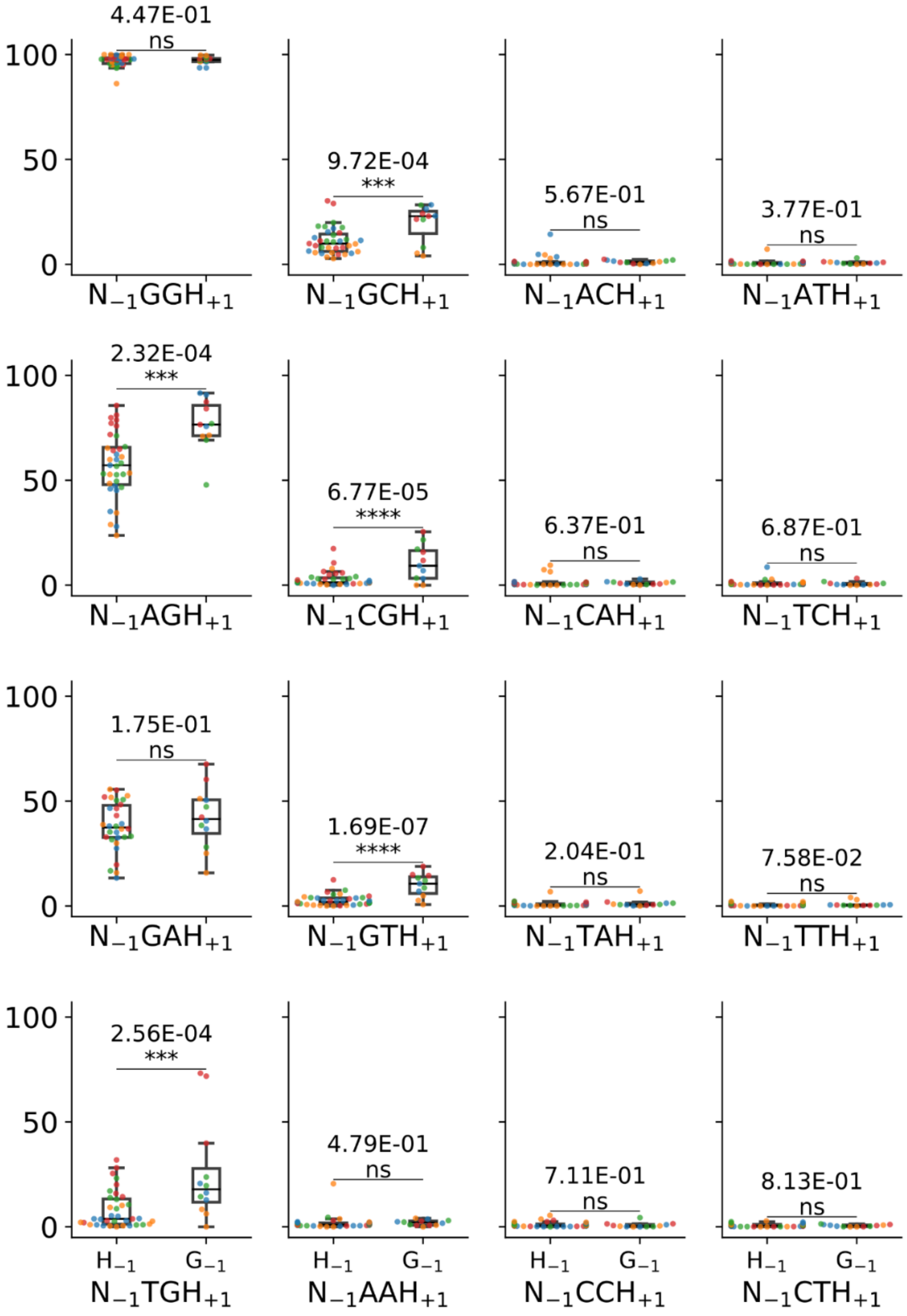

Supplementary Figure 14. Indel frequency at local sliding upstream PAMs in Dox+ HEK293T cells.

Same as **Supplementary Fig. 12** in cells treated with Doxycycline (Dox+). Number of elements in each box, from top to bottom, left to right: 35; 12; 35; 11; 30; 12; 36; 12; 35; 11; 35; 11; 36; 11; 34; 12; 35; 12; 35; 12; 31; 12; 35; 12; 36; 11; 35; 11; 36; 12; 35; 12.

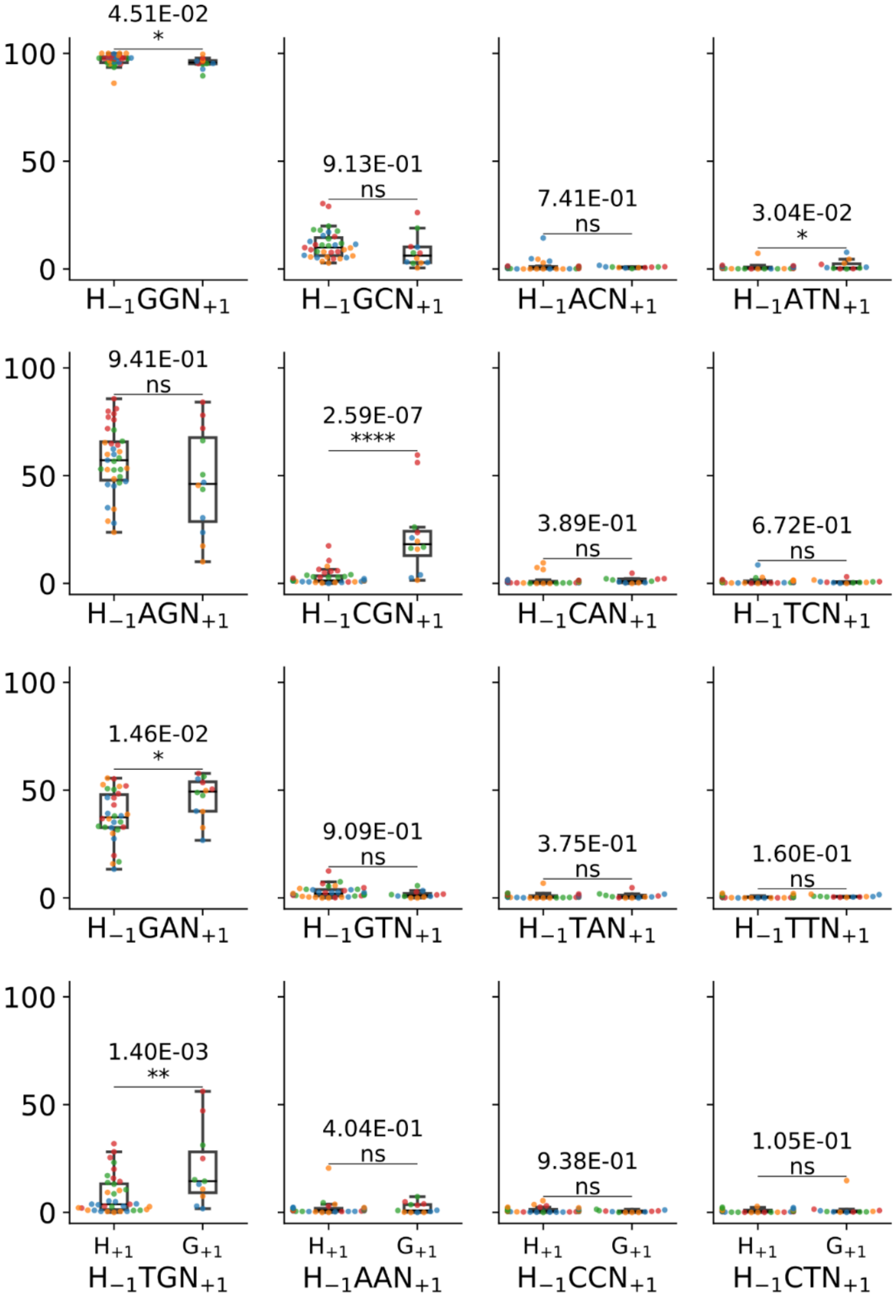

Supplementary Figure 15. Indel frequency at local sliding downstream PAMs in Dox+ HEK293T cells.

Same as **Supplementary Fig. 13** in cells treated with Doxycycline (Dox+). Number of elements in each box, from top to bottom, left to right: 35; 12; 35; 12; 30; 12; 36; 11; 35; 12; 35; 12; 36; 12; 34; 12; 35; 11; 35; 11; 31; 12; 35; 12; 36; 11; 35; 12; 36; 12; 35; 12.

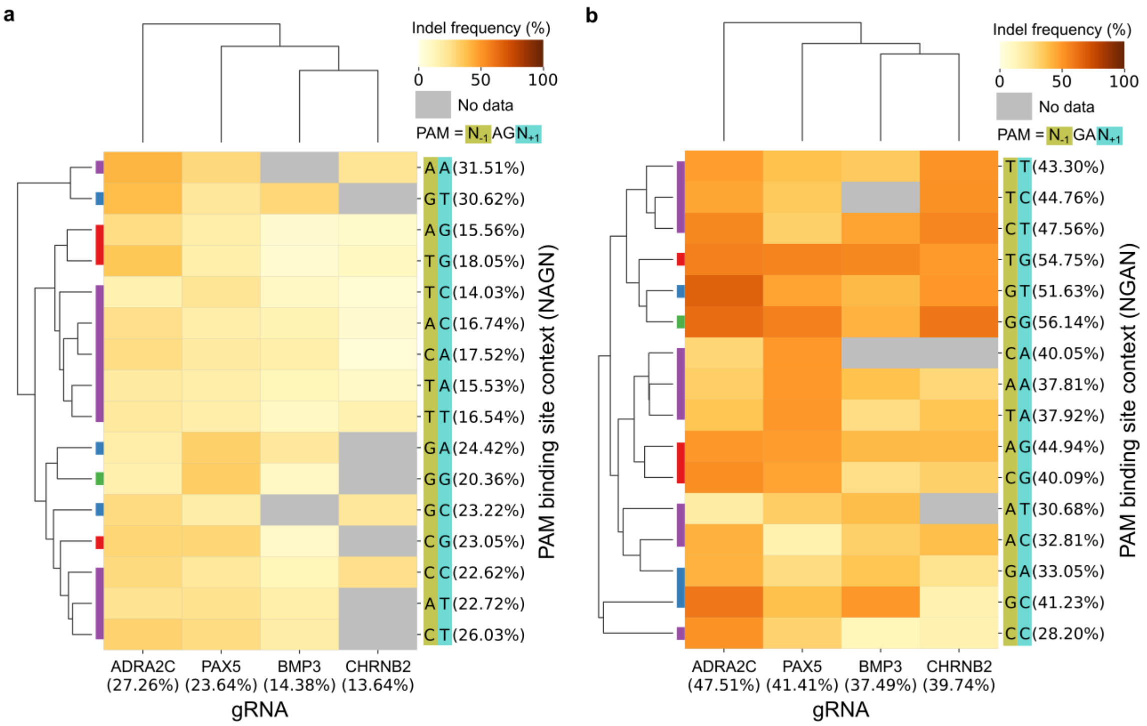

Supplementary Figure 16. Indel frequency at alternative PAM binding sites AG and GA with variable context in HEK293T cells.

(**a**) Heatmap of indel frequencies of 4 gRNAs targeting DNA sites with 5'-$N_{-1}AGN_{+1}$-3' PAMs clustered by $N_{-1}N_{+1}$ sequence, variated in all possible ways. Indel frequencies are measured in untreated (Dox-) cells. The hierarchical clustering is based on the Euclidean distance between vectors (rows). Grey boxes represent examples for which no data is available after pre-processing; for clustering, the missing values are linearly interpolated from targets with the same context. The averages of columns and rows are shown in parenthesis. Colours of the leaves in the left dendrogram (left) as in **Fig. 3**. **(b)** Same as **a** for 5'-$N_{-1}GAN_{+1}$-3' PAMs; indel frequencies measures in treated cells (Dox+).

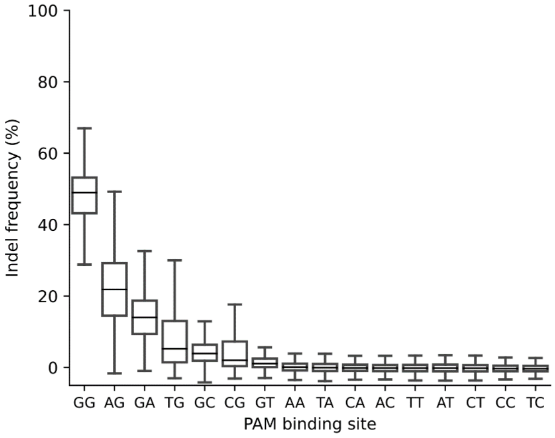

Supplementary Figure 17. Indel frequency of wild-type SpCas9 at PAM binding sites with variated sequences, Kim et al. (2020) dataset.

Indel frequency of gRNAs binding at DNA targets with different PAM binding sites (X-axis). For each of 30 gRNAs, the indel frequency at target DNA sites carrying different PAM contexts ($N_{-1}N_{+1}$ in $N_{-1}NNN_{+1}$ where the middle NN is the PAM binding site) was measured in HEK293T cells. Boxes are defined as in **Supplementary Fig. 1**. Number of gRNAs in each box, left to right: 470; 465; 466; 462; 461; 466; 468; 459; 443; 458; 461; 456; 453; 463; 469; 462.

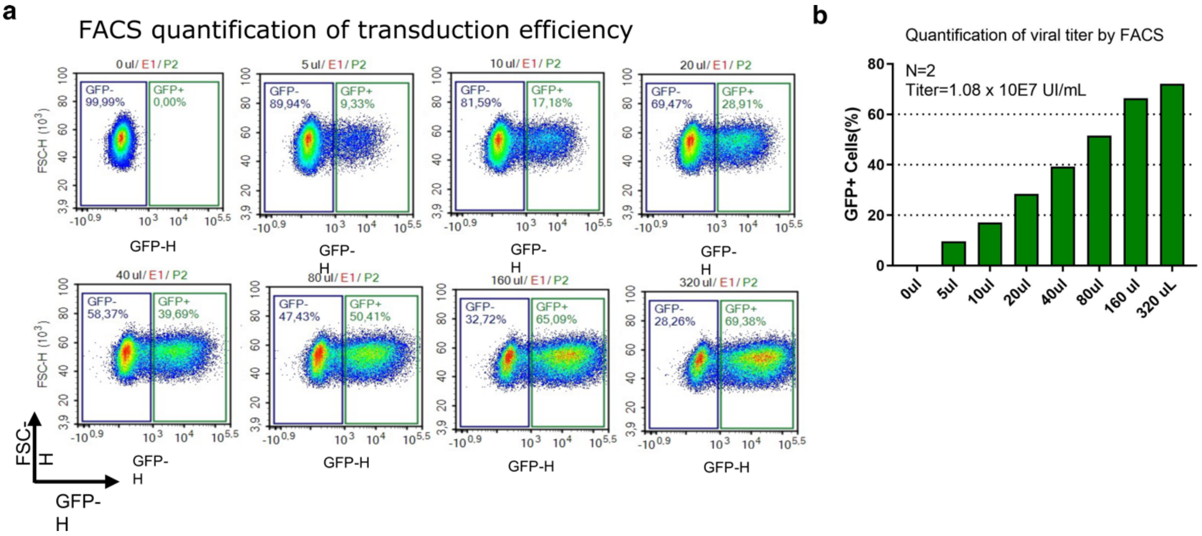

Supplementary Figure 18. Quantification of lentiviral library titer.

(**a**) Representative flow cytometry results (n=2) with gating for GFP positive and GFP negative cells and (**b**) quantifications of lentiviral functional transduction titer by quantifying mean GFP+ positive cells from duplicates. Volumes indicate the amount of crude lentivirus used per transduction.

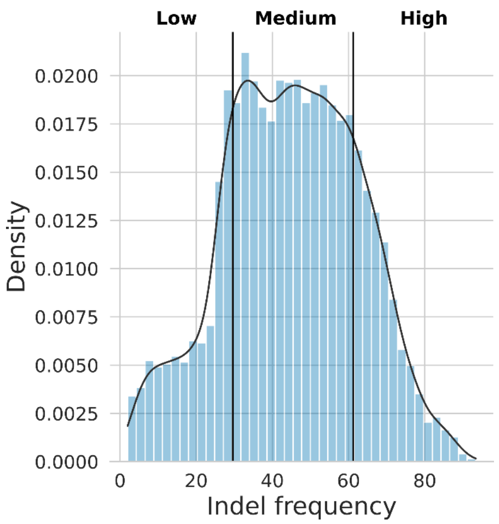

Supplementary Figure 19. Partition of gRNAs in efficiency groups.

The distribution of indel frequencies of 11,602 gRNAs from the merged Xiang et al. (2021) dataset (excluding the test portion) is divided in 3 partitions, which represent the efficiency sets Low (bottom 20%, *n*=2321), High (top 20%, *n*=2321) and Medium (remaining 60%, *n*=6960).

Supplementary Tables

Supplementary Table 1. Dataset pre-processing and filtering.

Number of gRNAs in the merged dataset of Xiang et al. (2021), and in the dataset of Hart et al. (2015) before and after pre-processing.

		Xiang et al. (2021)	Hart 2015
	Efficiency evaluation method	Indel freq.	knockout
	Num. initial entries (gRNAs+context)	23,902	4,239
Filter	Not present in hg38/no CRISPRoff output	23,096	4,238
	Not overlapping target CDS	-	4,184
	High sgRNA unpaired distance to optimal structure	21,941	4066
	Indel frequency <2%	21,402	-

Train-test splits and overlap	Split train-test by 30mer protospacer + context similarity	Train: 14,981	Test: 6421	-
	Remove 20mer gRNAs similar to examples in train set	-	6361	4026
Filter	CRISPRspec >= 5	11,602	-	-

Supplementary Table 2. Contingency table of hybridization free energy change. Contingency table of gRNAs split into efficiency groups, with rows representing intervals of hybridization free energy change ΔG_H to which gRNAs are assigned. Chi-squared contingency test (SciPy[1]) one-sided P=2.30E-67, degrees of freedom=2.

	Efficiency		
	Low	Medium	High
$\Delta G_H[-64.53, -47.09]$	1334	5054	1857
$\Delta G_H[(-\infty, -64.53) \cup (-47.09, +\infty)]$	987	1906	464

Supplementary Table 3. Contingency table of DNA-DNA binding free energy change. Contingency table of gRNAs split into efficiency groups, with rows representing intervals of DNA-DNA binding free energy change ΔG_O to which gRNAs are assigned. Chi-squared contingency test (SciPy[1]) one-sided P=4.00E-36, degrees of freedom=2.

	Efficiency		
	Low	Medium	High
$\Delta G_O[-31.81, -25.36]$	1512	5342	1857
$\Delta G_O(-\infty, -31.81) \cup (-25.36, +\infty)$	809	1618	464

Supplementary Table 4. Contingency table of minimum gRNA self-folding free energy change.

Contingency table of gRNAs split into efficiency groups, with rows representing intervals of minimum self-folding free energy change ΔG_U to which gRNAs are assigned. Chi-squared contingency test (SciPy[1]) one-sided P=1.24E-06, degrees of freedom=2.

	Efficiency		
	Low	Medium	High
$\Delta G_U[-3.30,0.00]$	1731	5288	1870
$\Delta G_U(-\infty,-3.30)$	590	1672	451

Supplementary Table 5. Contingency table of ΔG_B residual binding free energy change.

Contingency table of gRNAs split into efficiency groups, with rows representing intervals of ΔG_B residual binding free energy change to which gRNAs are assigned. Chi-squared contingency test (SciPy[1]) one-sided P=3.29E-107, degrees of freedom=2.

	Efficiency		
	Low	Medium	High
$\Delta G_B(-\infty,-21.90]$	1191	4969	1857
$\Delta G_B(-21.90,+\infty)$	1130	1991	464

Supplementary Table 6. Stacking base pair free energy change contributions.

Free energy change contribution of stacking base pairs in kcal/mol, modified from[2] to allow termination at bulged interactions, weighted as bulge extensions. The strandness is 5'-3' for the RNA query and 3'-5' for the DNA target. Rows represent the query's 1st of two neighbour nucleotides, columns the 2nd. Thus, the cell at row A and column A corresponds to an AA in the gRNA (and a TT in the DNA), and the reported free energy change refers to stacking an rA·dT on top of another rA·dT (r=RNA, d=DNA). See the Methods for more details on how the stacking base pairs free energy changes are derived.

Stacking 1st bp/2nd bp	A	C	G	U	-
A	-1.00	-2.10	-1.80	-0.90	0.25
C	-0.90	-2.10	-1.70	-0.90	0.00
G	-1.30	-2.70	-2.90	-1.10	0.00
U	-0.60	-1.50	-1.60	-0.20	0.25

-	-2.24	-2.49	-2.49	-2.24	+∞

Supplementary Table 7. Free energy change properties of gRNAs forming DNA-bulged gRNA-DNA interactions.

Free energy change properties and efficiency of gRNAs binding at targets with or without forming DNA bulges. Four gRNAs (R-01, R-30, R-08, and R-25) were evaluated by Lin et al.[3] and modified by removing 1 nt at each position on the gRNA to create DNA bulges in the gRNA-DNA hybrid. For each of the 4 gRNA, the top row shows the efficiency and the properties of the binding in which the full 20 nt gRNA was used, while subsequent rows show the interactions lacking one nt in the gRNA at different positions. Removed nucleotides are represented with dashes in the gRNA sequences. Only interactions with percentage indel of at least 10% in HEK293T cells are shown. Free energy changes are in kcal/mol and in bold if outside of the optimal free energy change range.

gRNA R-01 for *HBB* GUGAACGUGGAUGAAGUUGG (GC-content 50%)						
gRNA	% indel HEK293T	bulge-PAM distance	ΔG_H	ΔG_U	ΔG_O	ΔG_B
GUGAACGUGGAUGAAGUUGG	45	None	-50.49	0	-26.47	-24.02
G-GAACGUGGAUGAAGUUGG	40.1	19	**-45.38**	0	**-23.58**	**-21.80**
GU-AACGUGGAUGAAGUUGG	24.3	18	**-42.66**	-0.3	**-22.28**	**-20.08**
GUGAACGUGGAUG-AGUUGG	14.3	6-7	**-41.57**	-0.6	-26.47	**-14.50**
GUGAACGUGGAUGAAGUUG-	31.9	1-2	**-39.42**	0	-26.47	**-12.95**
gRNA R-30 for *CCR5* GUAGAGCGGAGGCAGGAGGC (GC-content 70%)						
gRNA	% indel HEK293T	bulge-PAM distance	ΔG_H	ΔG_U	ΔG_O	ΔG_B
GUAGAGCGGAGGCAGGAGGC	30	None	**-66.85**	-1.7	-30.02	-35.13
G-AGAGCGGAGGCAGGAGGC	44	19	**-63.45**	-1.7	-28	-33.75
GU-GAGCGGAGGCAGGAGGC	43.8	18	-60.27	-1.7	-26.72	-31.85
GUAGAGCGG-GGCAGGAGGC	53.4	11	-56.31	0	-30.02	-26.29
GUAGAGCGGA-GCAGGAGGC	28.4	10-9	-52.48	0	-30.02	-22.46
GUAGAGCGGAGG-AGGAGGC	40.8	8	-54.51	0	-30.02	-24.49
GUAGAGCGGAGGC-GGAGGC	22.1	7	-56.75	-1.6	-30.02	-25.13
GUAGAGCGGAGGCAGGA-GC	54.5	3-2	-51.47	-1.7	-30.02	**-19.75**
GUAGAGCGGAGGCAGGAGG-	32.1	1	-55.44	-1.7	-30.02	-23.72
gRNA R-08 for *HBB* GCUGUGGGGCAAGGUGAACG (GC-content 65%)						

gRNA	% indel HEK293T	bulge-PAM distance	ΔG_H	ΔG_U	ΔG_O	ΔG_B
GCUGUGGGGCAAGGUGAACG	25	None	-55.48	-2.7	-27.75	-25.03
G-UGUGGGGCAAGGUGAACG	13	19	-53.47	0	-26.47	-27
GC-GUGGGGCAAGGUGAACG	23.5	18	-50.67	-1.8	-25.02	-23.85
GCU-UGGGGCAAGGUGAACG	30.8	17	-48.08	-1.5	**-23.58**	-23
gRNA R-25 for *CCR5* GUGUUCAUCUUUGGUUUUGU (GC-content 35%)						
gRNA	% indel HEK293T	bulge-PAM distance	ΔG_H	ΔG_U	ΔG_O	ΔG_B
GUGUUCAUCUUUGGUUUUGU	27	None	**-33.6**	0	**-24.16**	**-9.44**

Supplementary Table 8. gRNA sequences selected to evaluate Cas9-mediated cleavage efficiency at surrogate target sites with different PAMs.

A total of 4 gRNAs were selected from the CRISPRon chip to evaluate Cas9-mediated cleavage efficiency at surrogate target sites with different PAM. For each gRNA the table reports its corresponding CRISPRon chip entry[4], the target gene name (used as ID for the gRNA), the sequence of the gRNA, the number of surrogates at which cleavage efficiency is evaluated and the indel frequency reported in dox+ HEK293T cells[4].

CRISPRon chip entry	Target gene	gRNA sequence	Surrogate number	Indel freq. (%)
1227	BMP3	UAUCACUCAACUCUUGAGGA	266	91.87%
2149	CHRNB2	GCGACGCCAGCGUGAGCGCG	265	93.20%
7673	PAX5	ACAGCAGCAGCUGGAGGUGC	265	98.98%
328	ADRA2C	UCACCGACCAGUAGCGGUCC	266	99.17%

References

1. Virtanen, P. et al. SciPy 1.0: fundamental algorithms for scientific computing in Python. *Nature Methods* **17**, 261-272 (2020).
2. Alkan, F., Wenzel, A., Anthon, C., Havgaard, J.H. & Gorodkin, J. CRISPR-Cas9 off-targeting assessment with nucleic acid duplex energy parameters. *Genome Biol* **19**, 177 (2018).
3. Lin, Y. et al. CRISPR/Cas9 systems have off-target activity with insertions or deletions between target DNA and guide RNA sequences. *Nucleic Acids Res* **42**, 7473-7485 (2014).
4. Xiang, X. et al. Enhancing CRISPR-Cas9 gRNA efficiency prediction by data integration and deep learning. *Nat Commun* **12**, 3238 (2021).
