# Supplementary material for: CRISPR/Cas9 gRNA activity depends on free energy changes and on the target PAM context
Source: Nat Commun. 2022 May 30;13:3006. doi: 10.1038/s41467-022-30515-0 (PMC9151727; doi:10.1038/s41467-022-30515-0)
Supplement: Supplementary file 2 — Description of Additional Supplementary Files [file 41467_2022_30515_MOESM2_ESM.pdf]

Description of Additional Supplementary Files

File Name: Supplementary Data 1
Description: Cas9-mediated gRNA cleavage efficiency data measured in HEK293T cells.
Cas9-mediated indel frequencies of 4 gRNAs on target DNA sites flanked by NNNN PAMs measured by deep sequencing in HEK293T cells 2-, 6-, and 10-days post transduction. Dox+ indel frequencies are also available for day 6 and 10. The data was processed by removing samples with total read count < 90 (dox-) or < 35 (dox+). Please see separate Excel file.

File Name: Supplementary Data 2
Description: List of oligonucleotides.
List of gRNA-target-DNA oligos employed in the random PAM library.
