# Supplementary material for: CRISPR/Cas9 gRNA activity depends on free energy changes and on the target PAM context
Source: Nat Commun. 2022 May 30;13:3006. doi: 10.1038/s41467-022-30515-0 (PMC9151727; doi:10.1038/s41467-022-30515-0)
Supplement: Supplementary file 5 — Reporting Summary [file 41467_2022_30515_MOESM5_ESM.pdf]

nature portfolio

Corresponding author(s): Yonglun Luo, Jan Gorodkin

Last updated by author(s): 2022/04/07

Reporting Summary

Nature Portfolio wishes to improve the reproducibility of the work that we publish. This form provides structure for consistency and transparency in reporting. For further information on Nature Portfolio policies, see our Editorial Policies and the Editorial Policy Checklist.

Statistics

For all statistical analyses, confirm that the following items are present in the figure legend, table legend, main text, or Methods section.

n/a	Confirmed	
☐	☒	The exact sample size (n) for each experimental group/condition, given as a discrete number and unit of measurement
☐	☒	A statement on whether measurements were taken from distinct samples or whether the same sample was measured repeatedly
☐	☒	The statistical test(s) used AND whether they are one- or two-sided<br>*Only common tests should be described solely by name; describe more complex techniques in the Methods section.*
☒	☐	A description of all covariates tested
☐	☒	A description of any assumptions or corrections, such as tests of normality and adjustment for multiple comparisons
☐	☒	A full description of the statistical parameters including central tendency (e.g. means) or other basic estimates (e.g. regression coefficient) AND variation (e.g. standard deviation) or associated estimates of uncertainty (e.g. confidence intervals)
☐	☒	For null hypothesis testing, the test statistic (e.g. F, t, r) with confidence intervals, effect sizes, degrees of freedom and P value noted<br>*Give P values as exact values whenever suitable.*
☒	☐	For Bayesian analysis, information on the choice of priors and Markov chain Monte Carlo settings
☒	☐	For hierarchical and complex designs, identification of the appropriate level for tests and full reporting of outcomes
☐	☒	Estimates of effect sizes (e.g. Cohen's d, Pearson's r), indicating how they were calculated

Our web collection on statistics for biologists contains articles on many of the points above.

Software and code

Policy information about availability of computer code

Data collection	No software was used.
Data analysis	Biopython v.1.77, Matplotlib v.3.2.2, NumPy v.1.18.5, Pandas v.1.2.0, Pysam v.0.15.0, Python v.3.8.3, Scikit-learn v.0.23.1, SciPy v.1.5.0 and 1.6.3, Seaborn v.0.11.1, RNAfold v.2.2.5, CRISPRoff v.1.1.2, RIsearch v.2.1, slogo v.1.1, FastQC v.0.11.3, fastp v.0.19.6, FLASh v.1.2.1, BWA-MEM v.0.7.17, FACSDiva v.9.0, NovoExpress v.1.5.6.<br>The RIsearch v.1.1 software was extended into RIsearch v.1.2 to allow the evaluation of RNA-DNA hybridization free energy changes as explained in the methods. These and other versions of the RIsearch software are available at https://github.com/RTH-tools/risearch/ and https://rth.dk/resources/risearch/.

For manuscripts utilizing custom algorithms or software that are central to the research but not yet described in published literature, software must be made available to editors and reviewers. We strongly encourage code deposition in a community repository (e.g. GitHub). See the Nature Portfolio guidelines for submitting code & software for further information.

Data

Policy information about availability of data

All manuscripts must include a data availability statement. This statement should provide the following information, where applicable:

- Accession codes, unique identifiers, or web links for publicly available datasets
- A description of any restrictions on data availability
- For clinical datasets or third party data, please ensure that the statement adheres to our policy

The raw sequencing data generated in this study have been deposited in the NCBI Sequence Read Archive under accession code BioProject: PRJNA732236 [https://www.ncbi.nlm.nih.gov/bioproject/PRJNA732236] and in the China National GeneBank under accession code CNP0001874 [https://db.cngb.org/search/project/

CNP0001874/]. The primary sequence of the human genome hg38 was downloaded from the NCBI, RefSeq assembly accession GCF_000001405.38 (https://www.ncbi.nlm.nih.gov/assembly/GCF_000001405.38/). Additional datasets employed in this study were obtained from the supplementary material of the following publications: dataset by Xiang et al. (2021) [https://doi.org/10.1038/s41467-021-23576-0], dataset by Kim et al. (2019) [https://doi.org/10.1126/sciadv.aax9249], dataset by Lin et al. (2014) [https://doi.org/10.1093/nar/gku402], dataset by Tsai et al. (2015) [https://doi.org/10.1038/nbt.3117], dataset by Hart et al. (2015) [https://doi.org/10.1186/s13059-016-1012-2], datasets by Kim et al. (2020) [https://doi.org/10.1038/s41587-020-0537-9]. Source data are provided with this paper.

Field-specific reporting

Please select the one below that is the best fit for your research. If you are not sure, read the appropriate sections before making your selection.

☒ Life sciences ☐ Behavioural & social sciences ☐ Ecological, evolutionary & environmental sciences

For a reference copy of the document with all sections, see nature.com/documents/nr-reporting-summary-flat.pdf

Life sciences study design

All studies must disclose on these points even when the disclosure is negative.

Sample size	No sample size calculation was performed. We found that the evaluation of SpCas9 cleavage efficiency on approx. 256 targets for each of 4 single guide RNAs was sufficient to validate, with statistical significance, the trends observed in a larger dataset of 11,602 gRNA-targets SpCas9 cleavage efficiencies. The latter was downloaded from publicly available sources and was previously shown to be appropriate for SpCas9 cleavage efficiency prediction.
Data exclusions	To increase the accuracy of the indel frequency, we removed gRNAs supported by less than 90 reads in the DOX free dataset and 35 reads in the DOX-induced dataset. These data exclusion steps were not pre-established.
Replication	All experiments were performed in at least duplicates and all attempts were successful.
Randomization	To test the improvement obtained in estimating Cas9-gRNA specificity after the introduction of local sliding, gRNAs were split randomly into train and test sets, keeping sequences with <4 differences in the 30mer (gRNA+target context) in the same subset to make the test set independent.
Blinding	Blinding was not relevant in this study, as the only group allocation (train/test dataset separation in the data analysis) was randomized computationally.

Reporting for specific materials, systems and methods

We require information from authors about some types of materials, experimental systems and methods used in many studies. Here, indicate whether each material, system or method listed is relevant to your study. If you are not sure if a list item applies to your research, read the appropriate section before selecting a response.

Materials & experimental systems

n/a	Involved in the study	
☒	☐	Antibodies
☐	☒	Eukaryotic cell lines
☒	☐	Palaeontology and archaeology
☒	☐	Animals and other organisms
☒	☐	Human research participants
☒	☐	Clinical data
☒	☐	Dual use research of concern

Methods

n/a	Involved in the study	
☒	☐	ChIP-seq
☐	☒	Flow cytometry
☒	☐	MRI-based neuroimaging

Eukaryotic cell lines

Policy information about cell lines

Cell line source(s)	The HEK293T was purchased from ATCC, catalog number CRL-3216
Authentication	The cell lines were not authenticated
Mycoplasma contamination	The cell line tested negative for mycoplasma contamination
Commonly misidentified lines (See ICLAC register)	No commonly misidentified cell lines were used in the study

Flow Cytometry

Plots

Confirm that:

☒ The axis labels state the marker and fluorochrome used (e.g. CD4-FITC).

☒ The axis scales are clearly visible. Include numbers along axes only for bottom left plot of group (a 'group' is an analysis of identical markers).

☒ All plots are contour plots with outliers or pseudocolor plots.

☒ A numerical value for number of cells or percentage (with statistics) is provided.

Methodology

Sample preparation	All cell lines used were treated with 4% formalin solution at RT for 20 min after harvesting
Instrument	BD LSRFortessa
Software	BD FACSDiva v.9.0
Cell population abundance	No cell sorting was performed. All population in flow cytometric analysis >1% in frequency are presented with their frequencies in the figures.
Gating strategy	Cells were first gated via FSC-H and SSC-H to define the main population (~80% of the population), and cells were next gated via FSC-H and FSC-A to define single cells (~90% of the population). Plots in the figures show all cells that pass these gates.

☒ Tick this box to confirm that a figure exemplifying the gating strategy is provided in the Supplementary Information.
